# Evaluation of the Wechsler Individual Achievement Test-Fourth Edition as a Measurement Instrument

**DOI:** 10.3390/jintelligence10020030

**Published:** 2022-05-22

**Authors:** A. Alexander Beaujean, Jason R. Parkin

**Affiliations:** 1 Psychology & Neuroscience Department, Baylor University, Waco, TX 76798-7334, USA; 2 Department of Teaching, Learning and Social Justice, Seattle University, Seattle, WA 98122, USA; parkinj@seattleu.edu

**Keywords:** validity, Wechsler Individual Achievement Test, test review, measurement, academic achievement

## Abstract

The Wechsler Individual Achievement Test (WIAT-4) is the latest iteration of a popular instrument that psychologists employ to assess academic achievement. The WIAT-4 authors make both pragmatic and measurement claims about the instrument. The pragmatic claims involve being useful for identifying individuals in certain academic achievement-related groups (e.g., specific learning disability). The measurement claims are twofold: (a) the instrument’s scores represent psychological attributes, and (b) scores transformed to standard score values have equal-interval properties. The WIAT-4 authors did not provide the evidence necessary to support the pragmatic claims in the technical manual, so we could not evaluate them. Thus, we limited our evaluation to the measurement claims for the composite scores. To do so, we used information in the technical manual along with some additional factor analyses. Support for the first measurement claim varies substantially across scores. Although none of the evidence is particularly strong, scores in mathematics and reading domains tend to have more support than the writing and total achievement scores. Support for the second claim was insufficient for all scores. Consequently, we recommend that psychologists wishing to interpret WIAT-4 composite scores limit those interpretations to just a few in the mathematics and reading domains. Second, psychologists should completely refrain from using any composite score in a way that requires equal-interval values (e.g., quantitative score comparisons). Neither of these recommendations necessarily disqualifies the scores from being useful for pragmatic purposes, but support for these uses will need to come from evidence not currently provided in the WIAT-4 technical manual.

Users of any psychological instrument have the burden of supporting their use of it (American Educational Research Association et al. 2014; Kline 1998). As such, it is critical that psychologists rigorously evaluate every instrument they employ (Mitchell 1984). It is often years after an instrument is published before peer-reviewed literature is available, so potential users wishing to make an instrument-adoption decision before then must rely on the information produced by the instrument authors.^1^ Thus, it is incumbent for instrument authors to provide sufficient information about the instrument for potential users to make an informed decision about whether to adopt the instrument (International Test Commission 2001). In this article, we review the fourth edition of the Wechsler Individual Achievement Test (WIAT-4; NCS Pearson 2020) and evaluate it using information provided in the instrument’s technical manual. Before doing so, we first discuss what is involved in evaluating psychological instruments.

## 1. Evaluating Psychological Instruments

The phrase “evaluating a psychological instrument” is somewhat of a misnomer because it does not involve evaluating an instrument itself as much as it involves evaluating (a) statements (claims) the instrument authors make about its intended uses, and (b) evidence (arguments) to support the truthfulness of those claims (Campbell et al. 2008; Kane 2013). As such, evaluations of psychological instruments should differ substantially based on the instrument’s purposes—something that is often “insufficiently recognized” (Ozer and Reise 1994, p. 363). We can class the purposes for most scientific instruments as measurement or pragmatic (Hand 2016; Lindquist 1936).

*Measurement purposes* are those that concern representation, specifically depicting an attribute’s manifestations and the relations among them (but see Michell 1999). Evaluating measurement claims involves evaluating the instrument’s validity (i.e., validation; Borsboom et al. 2004). *Pragmatic purposes* involve making decisions (e.g., provide treatment, make diagnoses), so evaluating pragmatic claims primarily involves evaluating evidence for the scores’ utility (e.g., sensitivity, cost-benefit). Pragmatic and measurement purposes are not mutually exclusive, so it is possible to employ an instrument’s scores for (a) only pragmatic purposes, (b) only measurement purposes, or (c) both pragmatic and measurement purposes (Newton 2017). Measurement and pragmatic uses are more or less independent of each other, however, so it is possible for an instrument’s scores to have strong utility evidence without measuring anything (or vice versa).

### 1.1. Validity

The concept of validity in the context of psychological measurement goes back to 19th century, but it did not become something of major interest to psychologists until the 20th century (Newton and Shaw 2014). Although validity quickly became an ambiguous concept in psychology (Slaney 2017), since the mid-20th century psychologists have increasingly employed it to mean something external to the instrument and contingent upon on particular interpretations of an instrument’s scores (e.g., Guilford 1946; Messick 1989). As such, support for validity claims is viewed as something discoverable through an ongoing process of assessing the correlations between an instrument’s scores and other phenomena (Reynolds 1998). This meaning of validity is troublesome (Markus and Borsboom 2013).

Pretend we have an instrument designed to measure people’s ability to add integers (i.e., *integer addition*). It may be interesting to know that the instrument’s score correlates with scores from other instruments—particularly instruments designed to measure integer addition. Two variables can correlate/not correlate for a variety of reasons,; however, only one of the two involves how well the scores represent integer addition (Borsboom 2005). Moreover, implicit in creating the instrument is some a priori knowledge about the meaning of the integer addition concept as well as the belief that the instrument’s score represents that concept (Krause 2005). Thus, correlations themselves cannot be the basis for determining whether the instrument measures integer addition (Guttman 1977, items 30–31). This does *not* entail that empirically acquired information is useless. To the contrary, empirical information is necessary to support certain claims about attributes needed to create a valid instrument (e.g., whether integer addition ability is a quantity; Mari et al. 2015). Likewise, empirical information can aid in selecting items from a pool of potential items that all cohere to the meaning of integer addition (Loevinger 1957) or spur further work in refining the integer addition concept (Krause 2012). Evaluating whether the instrument is valid, however, is a fundamentally a conceptual endeavor.

### 1.2. Evaluating the Validity of Psychological Instruments

Broadly, scientific instruments have validity to the extent that they measure the attributes they are designed to measure (Joint Committee for Guides in Metrology 2012). This entails that, for an instrument to be valid, (a) the intended-to-measure attribute has to exist as more than just a name (i.e., it has to be potentially measurable); and (b) variation in the attribute impinges on variation in score values the instrument produces (Borsboom et al. 2004). Although relatively straightforward, evaluating validity is not a simple endeavor—especially for instruments measuring psychological attributes. We will discuss a few components to such evaluations.

First, it is necessary to understand the meaning (i.e., rules for employment) of the to-be-measured attribute concept (Michell 2009). Most psychological attribute concepts are *functional*, so their rules for use involve things we do (Bem and De Jong 2013). Only psychological attributes whose meanings involve behavior are open to public observation, so those are the attributes we can ascribe to other people (Bennett and Hacker 2022; Coombs 1948).^2^ Being observable does not, however, guarantee measurability. Although we acknowledge there is not currently a consensus about the necessary or sufficient criteria for an attribute to be measurable (Mari et al. 2017), we believe the second and third components we discuss are necessary for measurability.

The second component is understanding how the behaviors that constitute a particular attribute go together. Like all other concepts, psychological attribute concepts are part of language, so psychologists are free to give them whatever meaning they want. As such, the behaviors criterial for a given attribute concept can go together a variety of ways, which are often not obvious. At one extreme are attributes whose behaviors go together because they have functional unity. If behaviors have *functional unity*, then they go together because of the behaviors themselves rather than the meaning of an attribute (Hearnshaw 1941; Peak 1953).^3^ In other words, the behaviors would still go together even if the attribute concept did not exist.

At the other extreme are attributes whose behaviors go together by fiat—they go together only because the concept includes them all. For example, psychologists often discuss *job morale* as if it is a single attribute, but the behaviors that constitute it (e.g., initiating activities, not seeking employment elsewhere, few absences) largely only go together because psychologists put them together when defining the job morale concept (Hardy 2009). Thus, it is not uncommon for employees to emit some of the behaviors but not others. To the extent this is true, representing job morale with a single score allows for the possibility of two people to be classified as having equal job morale yet manifest non-overlapping sets of behavior. This makes it difficult to support a claim that job morale is measurable. Two ways to rectify the situation are to restrict use of job morale to a hypernym for classifying job-related behaviors, or to make the meaning and representation of job morale multi-dimensional. Psychologists seldom employ either solution, however, but instead primarily look to study and measure attributes they can represent with a single score (Sijtsma 2006). In such cases, functional unity is a necessary condition for measurement.

Third, it is necessary to know the attribute’s different possible manifestations and the relations among the manifestations (e.g., equivalence, order, additivity) because this information determines whether an attribute is a quality, quantity, or something in between (Barrett 2018; Michell 2005). For example, it is self-evident that the integer addition ability has at least two manifestations: can add integers and cannot add integers. People can manifest the ability to add integers different ways, one of which is consistently responding to items about adding integers correctly. Likewise, one way people manifest not having the ability to add integers is consistently responding to integer addition items incorrectly. Since these two manifestations are mutually exclusive (i.e., it would be incoherent to state that the same person can both add integers and not add integers), we can represent the attribute on a so-called nominal scale.^4^ Of course, scientists do not rely on intuition for determining the different manifestations of an attribute and their relations. Instead, it is something that requires considerable conceptual and empirical work (Mari et al. 2015; Michell 1990).

Fourth, it is necessary to determine whether the instrument’s specifications (e.g., content, procedures) are consistent with what is currently known about the attribute (Krause 1967; Maraun 1998). For example, an instrument would not be valid for measuring the (overly simplistic) integer addition ability if it requires respondents to answer items such as “What is the capital city of Scotland?”, but could be valid if it had items such as “2 + 2 = ?”. Likewise, instruments producing scores with two values might represent the attribute faithfully (e.g., can/cannot add integers), but instruments producing more than two values (e.g., Normal Curve Equivalents) would not represent the attribute very well. Of course, it is not really the number of possible values that is important, but that all the known relations among attribute manifestations are faithfully represented in the relations among a score’s values.

## 2. Wechsler Individual Achievement Test–Fourth Edition

The WIAT-4 is multiple things simultaneously. It is (a) a standardized battery of individually administered instruments (i.e., subtests), each of which is comprised of items designed to elicit certain mental attributes and behavior; (b) a set of criteria for coding the elicited behavior; and (c) a set of algorithms for translating the coded behavior into values for different scores (i.e., scoring). As such, it is similar to many other academic achievement instruments currently available (e.g., Bardos 2020; Kaufman et al. 2014). The WIAT-4 is based on the third edition of the instrument (WIAT-3), but it is more than just an updated WIAT-3. The instrument authors not only collected data from a new norming sample, but also substantially added and revised items, subtests, and scores (Breaux 2020, p. 89). In addition, many of WIAT-4 scores are based on a psychological theory, which is notably different from the WIAT-3 wherein all the scores are atheoretical (Breaux 2020, pp. 89–96). As such, it is best to think of the WIAT-4 as a brand-new instrument rather than an update of a previously existing one (Beaujean 2015a; Bush et al. 2018).

### 2.1. Purpose of Wechsler Individual Achievement Test

The WIAT-4 authors claim the instrument can be used for both measurement and pragmatic purposes. They are explicit in their measurement claims, stating the instrument is “designed to measure the [academic] achievement of examinees ages 4 through 50, and students in prekindergarten (PK) through Grade 12” (Breaux 2020, p. 1; see also p. 28). In addition, the authors state that values of some of the scores “are on an equal-interval scale” (Breaux 2020, p. 64). Evaluating both claims require evaluating (measurement) validity evidence.

The pragmatic purposes involve using WIAT-4 scores for identifying members of various academic achievement-related groups (e.g., gifted, specific learning disability; Breaux 2020, pp. 83–87). Evaluating these claims involves evaluating empirical evidence about the scores’ utility. The utility evidence provided in the WIAT-4 technical manual consists of (a) basic descriptive statistics (e.g., means, standard deviation) of the scores for each group; (b) descriptive statistics for between-group score differences (e.g., standardized effect sizes); and (c) *p*-values for null hypotheses regarding mean differences between groups (Breaux 2020, pp. 47–60).^5^ While this information is somewhat useful, it is not sufficient for us to evaluate the scores’ utility (McFall and Treat 1999). Consequently, in our evaluation we focus exclusively on the evidence supporting the WIAT-4 authors’ measurement claims.

### 2.2. Wechsler Individual Achievement Test Scores

The WIAT-4 produces 32 scores (see Table 1), which we can classify different ways. One classification criterion is whether the score is comprised of other scores. *Simple scores* are those whose values are not dependent on the value of any other scores (i.e., based on a single set of items), while *composite scores* are those whose values are a function of simple scores. All WIAT-4 composite scores are unweighted sums of two or more simple scores (Breaux 2020, pp. 12–13). Most of the WIAT-4 subtests produce simple scores, but there are few exceptions (see notes in Table 1). A second criterion for classing scores is knowledge domain (i.e., content). The WIAT-4 authors designed the subtests’ items to elicit abilities in three core academic knowledge domains (i.e., reading, writing, mathematics) as well as in oral language (Breaux 2020, p. 28). All the WIAT-4 scores cover content from a single academic knowledge domain except for two: Total Achievement and Orthographic Processing.

The WIAT-4 authors state that interpreting the WIAT-4 scores should follow a four-step process (Breaux 2020, pp. 77–79).^6^

Step 1. Interpret the Total Achievement score.
Step 2a. Interpret all other composite scores and subtest scores normatively (i.e., compare how a respondent performed in reference to peers of the same age or grade).
Step 2b. Interpret all other composite scores and subtest scores ipsatively (i.e., compare scores within a single respondent).
Step 3. Identify ipsative strengths and weaknesses from composite scores. This involves (a) comparing each single-domain composite score for a respondent to the same respondent’s Total Achievement score, and (b) determining if the value difference is statistically different from zero.
Step 4. Make planned ipsative comparisons between different subtest scores or different composite scores. This involves (a) selecting multiple subtest or composite scores to compare, and then (b) determining if their value differences are statistically different from zero.

Implicit in the WIAT-4 interpretive guidance is the claim that each WIAT-4 score represents a distinct, although not necessarily unrelated, attribute. Consequently, it is necessary to evaluate the validity of each score. In this article, we focus on evaluating the evidence for the scores in steps 1 and 2a. We do so for two reasons. First, steps 2b–4 involve ipsative analysis and interpretation. *Ipsative* means “of the self”, so steps 2b–4 require comparing scores for a particular respondent to other scores for the same respondent (e.g., compare the Listening Comprehension score to the Reading Comprehension score; Cattell 1944). These interpretations are only warranted if the equal-interval claim is true. Second, although ipsative interpretations require certain measurement properties, they are primarily employed with the WIAT-4 for making pragmatic decisions (e.g., determining if a respondent has a psychological disorder or disability). Third, evaluating subtests entails evaluating their items, but the WIAT-4 authors provide little information about items in the technical manual. Although withholding this information from consumers became common practice in the mid-20th century, it is a lamentable practice because it precludes evaluation from disinterested scholars of interest (Buros 1977; Merton 1968).

## 3. Evaluation of the Wechsler Individual Achievement Test *Total Achievement Score*

The WIAT-4 authors state that the Total Achievement score “provides a measure of overall academic achievement in the areas of reading, math, and writing” (Breaux 2020, p. 113). Consequently, the first step in evaluating the validity of the Total Achievement score is understanding the meaning of the *overall academic achievement* (OAA) concept. Unfortunately, OAA is not a technical concept within either the psychology or education disciplines (i.e., it has no consistently shared meaning), and the WIAT-4 authors do not provide a definition. Thus, we need to explore the concept in more depth.

### 3.1. Meaning of Overall Academic Achievement

Psychologists have used OAA and similar terms for over a century, such as: general educational ability (Burt 1917), verbal-educational ability (Vernon 1950), scholastic achievement (Carroll 1943), schooling (French 1951), general academic intelligence (Dailey and Shaycoft 1961), and general academic achievement (Kaufman et al. 2012). With few exceptions, psychologists do not provide definitions or discuss the concepts’ meanings except for stating it is distinct from, but related to, what Charles Spearman (1927) called *g*. In doing so, psychologists assume readers already understand the concepts, which means psychologists are likely employing ordinary language meanings. Although ordinary language concepts are not uncommon in psychology, they can be troublesome because they are often vague or ambiguous (Vygotsky 1987) which makes evaluating validity a particularly challenging endeavor (Haynes et al. 1995). Consequently, instead of understanding the meaning of OAA by working through a technical definition, we have to take a different tack. Specifically, we must (a) work through how psychologists employ the concepts of *overall*, *academic*, and *achievement* (i.e., conceptual analysis; Hacker 2020); and then (b) reference those meanings to how the WIAT-4 authors discuss OAA and the procedures they use to measure it. Since the *overall* and *academic* terms modify *achievement*, we begin our conceptual work with achievement.

#### 3.1.1. Meaning of *Achievement*

The unmodified *achievement* concept has a family of related meanings (Achievement 2021), but we will just focus on the two that psychologists seem to employ the most. One meaning is as a conative concept involving the desire to do things in such a way that they meet some standard (e.g., Heckhausen 1967). We manifest this need or motivation for achievement by doing things we believe will either avoid disapproval or attain approval from ourselves or other persons (Crandall 1963). Psychologists have created different techniques and instruments to capture this form of achievement (e.g., projective testing, self-reports), but they all have in common coding respondents’ behavior using some criteria other than correctness.

A second meaning of achievement is as the production of a particular outcome, either tangible (e.g., a loaf of bread) or intangible (e.g., goodwill from others). More specifically, it is an instantaneous and relatively durable effect of our behavior on situations (Vendler 1957). This meaning is intertwined with our knowledge and abilities to use knowledge, so is more of an intellective concept than conative (Reeve and Bonaccio 2011). As such, the techniques and instruments psychologists have created to capture this meaning of achievement commonly require coding behavior based on correctness (Guttman and Levy 1991).

Some psychologists claim that intellective achievement is a process more than an outcome (e.g., Bradford 2016; Coffman 1970), but this is likely better captured by the accomplishment concept. An *accomplishment* is a kind of goal-oriented process such that reaching the intended goal justifies employing the accomplishment term (Stokes 2008). That is, accomplishments are purposeful processes that culminate in something (i.e., an achievement). For example, if Pedro wrote a novel, it would be an accomplishment because writing a novel is something people have to commit to doing. The instant his novel is published, however, it is an achievement.

The distinction between achievement and accomplishment may appear trivial, but it is important (Varzi and Torrengo 2006). Achievements *can* be the culmination of a process designed to result in the achievements, but they can also result from a series of accidental or haphazard events. Accomplishments, however, cannot be accidental or haphazard. By definition, they are intentional culminations so depend on (a) knowledge about how to produce some achievement, and (b) the ability to employ the knowledge in such a way as to culminate in the particular achievement. Thus, Kiko responding to the item “3 + 2 = ?” correctly is an accomplishment only if she did so by employing her integer addition knowledge, but is an achievement irrespective of whether she employed her integer addition knowledge, guessed, or used some other process.

#### 3.1.2. Meaning of *Achievement* in the Wechsler Individual Achievement Test

The scoring criteria for coding all responses to WIAT-4 items concern correctness, so we can deduce the instrument’s authors employ the achievement concept in a way that is more consistent with the intellective meaning than the conative one. In addition, they employ the concept more consistent with an instantaneous outcome than a process. It is true that the authors discuss the mental processes they believe respondents should employ when answering items within a particular subtest, but this information was only used for item creation and designing procedures for WIAT-4 users to conduct a demand analysis (Breaux 2020, pp. 61–63). The actual mental processes respondents employ in their item responses are neither elicited or coded as part of the WIAT-4 administration nor used in the scoring procedures.

#### 3.1.3. Meaning of *Academic* with Respect to *Achievement*

The unmodified achievement concept has a wide meaning and encompasses a variety of behaviors. As such, it is more a class of psychological attributes (i.e., umbrella concept) than a particular attribute. To limit the concept’s boundaries, psychologists add a variety of modifying terms (e.g., athletic, occupational), but we only focus on the academic modifier. The *academic* concept has a few different meanings, but they are closely interwoven and all relate to school or education (Academic 2021). Thus, *academic achievements* are achievements that people manifest either in formal educational settings or result from abilities acquired from knowledge typically taught as part of formal education (Ebel and Frisbie 1991). This is still a very wide concept, including everything from alphabetic letter knowledge to diagnosing a complex medical disorder correctly. Thus, psychologists typically take one of two tacks to further constrain the concept (Spinath 2012).

First, psychologists employ more domain-constraining modifiers (e.g., biochemistry achievement, nursing achievement). Psychologists typically do this when discussing achievements involving knowledge or abilities tied to particular curricula, so instruments designed to assess these achievements are also tied to curricula (e.g., curriculum-based assessments, licensing exams). Second, psychologists constrain the academic achievement concept to mean basic competencies typically acquired by members of a particular society or across multiple societies at certain ages. These competencies usually involve reading, writing, and using mathematics (Burt 1917; Mather and Abu-Hamour 2013). They are not tied to any particular curriculum, however, because psychologists create the instruments (a) to capture attributes that have some universality, and (b) for use with most or all societal members (Norenzayan and Heine 2005).

#### 3.1.4. Meaning of *Academic Achievement* in the Wechsler Individual Achievement Test

The WIAT-4 authors do not discuss any particular curricula, but do discuss how differences in respondents’ curriculum exposure can cause interpretational difficulties of some WIAT-4 scores (Breaux 2020, pp. 68, 72). Moreover, the Total Achievement score is comprised of scores from subtests in the reading, writing, and mathematics domains (see Table 1). Thus, we can infer that the WIAT-4 authors employ the academic achievement part of OAA to mean certain competencies members of American societies are expected to acquire.

#### 3.1.5. Relation between Academic Achievement and Intelligence Instruments

If an instrument that captures academic achievement is not tied to any particular curriculum, captures somewhat universal abilities, and applies to most-or-all members of a society, then this naturally raises the question of how academic achievement instruments relate to intelligence instruments. Psychologists have a long history of discussing academic achievement and intelligence instruments as being distinct kinds (e.g., Matsumoto 2009). This is because psychologists have traditionally viewed academic achievement and intelligence as being distinct kinds of attributes (Anastasi 1984). Intelligence comprises a person’s aptitude or potential to learn, while academic achievement is what a person has actually learned. The traditional view is flawed (Anastasi 1980; Wesman 1956). Support for this claim comes from the defining features of intelligence and intelligence instruments.

*Intelligence* is an ordinary language concept whose meaning has changed over time and geography (Goodey 2011; Spearman 1937). It entered the discipline of psychology in the 19th century by way of evolutionary biology (Danziger 1997). Biologists employed the concept as if it was a single attribute more or less synonymous with adaptive behavior or behavior flexibility. Psychologists tended to follow the biologists lead and employ the concept as if it was a single attribute, but not necessary one involving behavior flexibility/adaptation (cf. Bascom 1878; Taine 1872). Thus, there was ambiguity in the concept from the beginning.

Instead of reigning in the concept’s meaning, however, psychologists in the early 20th century loosen it via their various idiosyncratic employments (e.g., Rugg 1921).^7^ The concept eventually got so muddled that it became “a mere vocal sound, a word with so many meanings that finally it has none” (Spearman 1927, p. 14). One solution to this problem has been to re-define intelligence in such a way as to incorporate multiple existing meanings (e.g., Wechsler 1975). The major difficulty with this solution is that the resulting concepts are typically too vague to be measurable. A second solution is to invent new concepts that have a particular meaning and, often, a unique name (i.e., neologisms). Perhaps the best-known example is Spearman’s invention of the *g* concept. Importantly, he did so with the intention of creating a technical concept amenable to scientific investigation, not to redefine intelligence (e.g., Spearman 1927, 1933, 1938). Thus, the major difficulty with this solution is that it does not address the ambiguity of the intelligence concept.

A third solution is to employ intelligence as an umbrella concept capturing a class of related attributes rather than one particular attribute (Howard 1993). This was how Spearman employed the concept (e.g., Spearman and Wynn Jones 1950), as did many of his protégés (e.g., Cattell 1987). This tradition continues today, with a recent conceptual study of intelligence concluding that intelligence “is a generic term, which encompasses a variety of constructs and concepts” (Reeve and Bonaccio 2011, p. 188). A major issue with this third solution is determining the criteria for an attribute to be included or excluded. Although psychologists have discussed multiple criteria, it appears that all intellective attributes share at least three major features (Burt 1944; Hacker 2013).

First, they involve our abilities to do something rather than mental states, dispositions, or attitudes. Second, these abilities involve acquiring or employing knowledge more than bodily movement (i.e., physical attributes), feelings/emotions (i.e., affective attributes), or motivation/volition (i.e., conative attributes). We discussed earlier that both features apply to academic achievement competencies as well. That is, psychologists tend to use the academic achievement concept to mean a class of abilities involving the employment of knowledge typically acquired in formal educational settings (Monroe et al. 1930).

Third, the abilities exist on a spectrum (Carroll 1993). When discussing intellective attributes, psychologists typically discuss this spectrum by referencing the breadth of tasks that elicit the attribute. At one end of the spectrum are *specific abilities* that people employ for a narrow set of tasks, while at the other end are *broad abilities* that people employ for a wide variety of tasks. In the context of academic achievement attributes, psychologists discuss the spectrum by referencing the specificity of a knowledge domain (Reeve and Bonaccio 2011). At one end of this spectrum is *domain*-*specific knowledge* that has a very circumscribed applicability (e.g., history of Leeds, England), while at the other end is *domain-general knowledge* that has much wider applicability (e.g., how to construct a valid argument).

Domain-specific knowledge and specific intellective abilities are not exchangeable concepts, but they are not unrelated either (and likewise for domain-general knowledge and broad intellective abilities). Instead, they represent differences in emphases (Reeve and Bonaccio 2011). Thus, it is better to think of the academic achievement and intelligence concepts as differing in degree more than in kind (Anastasi 1984; Cronbach 1990). That is, they are abilities that exist on a spectrum ranging from involving specific knowledge applicable to a very narrow range of tasks to those involving more general knowledge applicable to a broad array of tasks (Anastasi 1976; Carroll 1993; Schneider 2013).

Since intellective attributes all share some common features, it is not surprising that the multiplicity of intelligence instruments also shares a set of features (Guttman and Levy 1991). These instruments (a) contain items that elicit specific behavioral responses from examinees; (b) require examinees exert maximal effort in responding to items; and (c) provide guidelines for coding responses based on satisfying some logical, factual, or semantic rules (i.e., correctness). These features apply to academic achievement instruments as well (Thorndike and Thorndike-Christ 2010). Thus, irrespective of whether psychologists use the term intelligence or academic achievement in an instrument’s name, the instrument measures (or potentially measures) the strength of one or more abilities a respondent has developed and is willing to demonstrate (Anastasi 1976, pp. 399–400).

#### 3.1.6. Meaning of *Overall*

*Overall* is a somewhat ambiguous concept that can mean everything (i.e., end to end), operating over an entire range of things, or taking everything into consideration. The WIAT-4 authors provide some help narrowing the meaning because they use the term *general academic achievement* as a synonym for OAA (Breaux 2020, p. 42). Thus, we can infer that they believe the overall and general concepts are interchangeable. Unfortunately, *general* is not exceptionally clear in its meaning. In psychology, it has at least three meanings: breadth, depth, and summary (Beaujean 2015b; Spearman 1927).

As *breadth*, general concepts have more elements (i.e., broader) than more specific (i.e., narrow) concepts. In measurement models, this relation is often represented by a bi-factor structure whereby the *indicators* (i.e., recorded observations of phenomena, such as items or subtests) are specified to be the effects of (i.e., result from) both broader and narrower attributes operating more or less independently of each other (Holzinger et al. 1937). As *depth*, general concepts are at a higher level (i.e., super-ordinate) than more specific (i.e., sub-ordinate) concepts. In measurement models, this relation is often represented by a higher-order factor structure whereby (a) a set of indicators are specified to be the effects of multiple related attributes; and (b) those attributes are specified to be the unobserved (unmeasured) effects of more super-ordinate attributes.

As *summary*, general concepts and specific concepts both condense information with the difference being that general concepts condense over wider content than specific concepts. This relation can be represented by models with a formative-indicator structure (e.g., weighted average) or causal-indicator structure (Bollen and Bauldry 2011). Either way, the indicators are specified to influence the attributes rather than the attributes influencing the indicators. This entails that indicators define the attributes, so changing indicators can alter what instruments capture. This is not troublesome for instruments designed for pragmatic purposes (i.e., making diagnostic decisions) because authors create such instruments to produce scores that consistently predict some criteria external to the instrument (Burisch 1984). Having indicators define attributes is troublesome for measurement instruments, however, because it runs counter to the measurement process in science (Edwards 2011). Scientific measurement requires specifying an attribute’s meaning before creating an instrument, which entails the meaning be invariant across indicators (Mari et al. 2015). Thus, it is unlikely that summary models are measurement models (Rhemtulla et al. 2015).

#### 3.1.7. Meaning of *Overall (General)* in the Wechsler Individual Achievement Test

The WIAT-4 authors likely do not employ the overall/general concept to mean depth because they do not discuss OAA as influencing more narrow attributes (e.g., reading fluency). The authors are more equivocal about the breadth and summary meanings. On the one hand, they imply a summary meaning when they state the Total Achievement score provides “a midpoint for determining the examinee’s relatively strong and weak areas of achievement” (Breaux 2020, p. 77). On the other hand, they imply a breadth meaning when they state the Total Achievement score provides an “overview of the examinee’s overall achievement” and should be interpreted in a manner consistent with all the other WIAT-4 scores (e.g., report the score, confidence interval, and percentile rank; Breaux 2020, p. 77). Since the WIAT-4 authors are unclear about their meaning of overall/general, we will assume they mean having more breadth and, thus, consider whether OAA is a potentially measurable attribute.

### 3.2. Evidence for Functional Unity

Our brief conceptual analysis allows us to state that the WIAT-4 authors likely employ the OAA concept to mean a complex psychological attribute that involves employing abilities constitutive of reading, writing, and using mathematics. Reading, writing, and using mathematics all manifest in certain behaviors, which means OAA is observable, but may or may not be measurable. A necessary condition for OAA to be measurable is that the behaviors that constitute it have functional unity. We introduced the functional unity concept earlier but will expand upon it here.

A set of behaviors has functional unity when they are related in such a way that if any one of them changes, then the others “suffer the same fate” (Cattell 1956, p. 69). One line of evidence supporting functional unity comes from empirical investigations. Specifically, designing experiments to evaluate whether a set of behaviors “rise together, fall together, appear together, disappear together or, in general, covary together” (Horn 1972, p. 161). Empirical evidence is not sufficient, however, because behaviors could go together for reasons other than an attribute having functional unity (Coombs 1948). Thus, in addition there needs to be a theory that provides a sound explanation for why the behaviors constitutive of a concept should hang together.

An example may clarify things. Pretend we have a battery with two subtests, both of which require respondents to listen and provide an oral response. One subtest contains items of the form “1 + 2 = ?”, while the other contains items of the form “Do you believe that you often have to rushed to complete school work?”. If we were to administer the battery to a set of elementary school students, it is not improbable that we would find that scores for the two subtests correlate at a level statistically different than zero (Lykken 1968). Although this corroborates the functional unity hypothesis, the unity is likely superficial because there is no theory explaining why behaviors across the subtests would go together. Instead, non-zero correlations among the subtests likely result from both subtests’ items having a common administration medium, response modality, and requiring respondents to remember information.

If performance on all the integer addition items involve employing the same attribute or set of attributes, then it is possible that the unity of integer addition behavior may go beyond the superficial to a causal construct. This should manifest in particular relations among item performances across people on a single occasion as well as within the same people across multiple occasions (Horn 1963; Zimprich and Martin 2009). For example, if the integer addition items are arranged in order of increasing difficulty, then we would expect that for all respondents who correctly answered item *p,* then the probability of the same respondents answering items 1, 2, …, *p* − 1 correctly is ≈1.00 (Loevinger 1947). Likewise, if we intervene with a particular student’s integer addition skills, then not only should the student be able to answer item *q* (*q* > *p*) correctly, but also be able to answer items 1, 2, …, *q* − 1 correctly as well. A possible explanation of this functional unity comes from the fact that mathematics is largely a graduated knowledge domain, so the ability to use more fundamental mathematics knowledge (e.g., adding integers without carrying) is usually necessary before being able to understand and use more advanced knowledge (e.g., adding integers with carrying).

#### 3.2.1. Empirical Evidence for Functional Unity

The WIAT-4 technical manual provides two sources of empirical evidence concerning functional unity of OAA. The first is a study in which the WIAT-4 authors investigated the relation between Total Achievement score values across time (i.e., 12–87 days) for a subset of the norming sample (Breaux 2020, pp. 20–24). If OAA has functional unity, then we should observe relatively large correlations values among the Total Achievement scores across such a relatively short period of time. The correlation values are indeed large (i.e., .93–.95), which corroborates the hypothesis of OAA having functional unity.

The second source of evidence is the correlations among the WIAT-4 subtests for the norming sample. If OAA has functional unity, then we should observe relatively strong correlations among the subtests that comprise Total Achievement score. The WIAT-4 authors support functional unity by relying on visual inspection of the correlations (Breaux 2020, p. 29), but this is subject to the same cognitive biases as other visual inspection of data. A more robust approach is to subject the correlations to a factor analysis (Loehlin and Beaujean 2016a). Since the WIAT-4 authors do not provide any factor analytic results, we conducted our own.

##### Factor Analysis of Wechsler Individual Achievement Test Norming Data

Data for the factor analyses came from the WIAT-4 norming sample, which consists of 1832 participants aged between 4 and 50 years and was stratified to be consistent with the 2018 U.S. Census information. The sample includes 120 participants for each year from age 4 to 16 years, 120 participants for the combined age range of 17–19 years, 100 participants between the ages of 20 and 30 years, and 52 participants between the ages of 31 and 50. All data was collected between October of 2018 and February 2020—before American schools closed due to the COVID-19 pandemic.

For all factor analyses, we used the subtest correlation matrices provided in the WIAT-4 technical manual (Breaux 2020, pp. 31–34). The technical manual provides combined correlation matrices for the following age groups: 4–7 years (*n* = 480), 8–11 years (*n* = 480), 12–19 years (*n* = 720), and 20–50 years (*n* = 152). Some of the subtest scores are composite scores because they are comprised of two or more component scores. For the Listening Comprehension, Oral Expression, and Sentence Composition subtests, we included the composite score in the correlation matrix instead of the individual component scores.

For all factor analyses, we employed an unconstrained (i.e., “exploratory”) model and used the entire correlation matrix rather than sets of particular subtests. We used the R statistical programming language (R Development Core Team 2017), particularly the *EFAtools* package (Steiner and Grieder 2020). Before initiating the factor extraction process, we subjected each correlation matrix to the Kaiser–Meyer–Olkin (KMO) test for sampling adequacy. KMO values were above .79 for each correlation matrix, so all matrices appear suitable for factor analysis.

To determine the number of factors to extract, we examined Kaiser’s criterion method (Kaiser 1974), minimum average partial test (MAP; Velicer 1976), and parallel analysis (Horn 1965). The results are given in the right part of Table 2. The MAP test routinely suggested the presence of three factors, eigenvalues derived from the subtest correlation matrices ranged from 3 to 4, and parallel analysis suggested five factors for all but the older age-group, where it suggested three factors. To gain additional clarity about the number of factors to extract, we used statistical measures of the goodness of fit for models with 3–5 extracted factors. The statistical indices were used are: χ^2^ goodness-of-fit test, Akaike information criterion (AIC), Bayesian information criterion (BIC), root mean square error of approximation (RMSEA) and comparative fit index (CFI). The χ^2^ goodness-of-fit test indicted that none of the models fit the data well for any of the age groups. The other fit indices indicated more factors produced increasingly better fit, although the change from the three- to the four-factor solution was noticeably larger than from the four- to five-factor solution.

Given the ambiguity of the criteria for choosing the number of factors, we extracted 3–5 factors for each of the correlation matrices using the principal axis technique. We rotated the factors using a bi-factor rotation (Jennrich and Bentler 2011).^8^ We did so because it allows for a general factor (representing OAA) and multiple non-overlapping group factors that possibly represent more specific attributes. We conducted the bi-factor rotation using the procedures described by Loehlin and Beaujean (2016b) using 1000 random starting values and retaining the 10 best solutions. When the analysis returned multiple solutions, we retained the one with the lowest minimization value. When interpreting the loadings, we considered .3 to be a lower bound for a salient loading.

The results from our factor analysis indicate that the subtests that comprise Total Achievement score do tend to form a breadth factor (Table A3, Table A5 and Table A7 in Appendix A). Across factor extractions and within each age group, all factor loadings on the general factor are above the salience criterion and are in same direction. At the same time, the factor loadings for some of the subtests appear to change noticeably across the age groups, especially for the oldest age group (20–50 years). For instance, in the solution with five specific factors, Essay Composition’s general factor loading appears to drop substantially between the 12–19 and the 20–50 age group. This is currently just a hypothesis, however, because a rigorous evaluation of invariance is well beyond the scope of this article. Thus, we can state that there is some empirical evidence corroborating functional unity of OAA within an age group, but it is unknown if the unity exists across age groups.

#### 3.2.2. Theoretical Evidence for Functional Unity

The technical manual contains no theoretical rationale for why the subtests that comprise OAA (as captured by the Total Achievement composite score) should hang together, much less a rationale for why some subtests might lose strength as indicators in adult respondents. Thus, we examined the intelligence and academic achievement literature for possible theories. One we believe is particularly useful is *triadic theory* (Cattell 1987; Cattell and Johnson 1986).^9^

In triadic theory, so-called crystallized intelligence (*g_c_*) represents our cumulative knowledge across all knowledge domains. Triadic theory’s investment aspect metaphorically explains *g_c_* as resulting from the investments of our broader intellective attributes (e.g., memory, fluid intelligence), conative attributes (e.g., interests), and formal and informal educational opportunities. In school-age children, *g_c_* often appears to be unitary across people, but this is not because *g_c_* has functional unity. Instead, it is an artifact of strong developmental and situational constraints (e.g., similar interests, similar school curricula). Once the constraints weaken, *g_c_* begins to differentiate (dissociate) into more specific attributes comprised of more specific knowledge (e.g., vocational, avocational).

To the extent that OAA and *g_c_* are the same or strongly overlapping concepts, we would expect that the factor loadings for the subtests that comprise the Total Achievement score would weaken across age, especially in adulthood. This is because schooling is compulsory in the United States until the beginning of emerging adulthood (approximately 18 years of age). The fact that major differences in the WIAT-4 factor loadings are more or less confined to the oldest age group is consistent with predictions from the investment theory aspect of triadic theory. Of course, there could be other explanations that are just as consistent with the observed factor loadings. Until such explanations are put forth, however, we do not believe there is a theory-based justification for believing that OAA has functional unity. As such, it is not measurable and, thus, the Total Achievement score cannot have measurement validity.

## 4. Other Composite Scores

Step 2a in the WIAT-4 score interpretation guidance involves interpreting the other composite scores. We focus only on the composite scores in the domains of reading, writing, and mathematics because the WIAT-4 authors state that the fourth domain (i.e., oral language) is “not a core area of achievement” (Breaux 2020, p. 114).

### 4.1. Reading

The WIAT-4 authors created the reading domain subtests to align with the *simple view of reading* theory and its extensions (Hoover and Gough 1990; Kilpatrick 2015). The simple view of reading explains reading achievement as resulting from two conceptually independent mental attributes: word decoding/reading and oral language/linguistic comprehension. *Word decoding/reading* is the ability to apply knowledge of the relations between printed language and spoken language. It requires *cipher skills* (i.e., knowledge of letter–sound correspondences) and *word-specific knowledge* (i.e., applying cipher skills to particular words). *Oral language/linguistic comprehension* is the ability to apply knowledge of the oral language in which the words are written. Later extensions of the simple view of reading include contextual reading fluency as a bridge concept linking word decoding/reading and oral language/linguistic comprehension with reading comprehension. *Contextual reading fluency* is the speed at which we can accurately read connected text.

The WIAT-4 provides multiple subtests designed to capture word decoding/reading along with composite scores for cipher skills and word-specific knowledge (see Table 3). The three cipher skills composite scores are: Basic Reading, Decoding, and Phonological Processing. *Basic Reading* is “a composite score that closely aligns with the definition of basic reading skills specified by IDEA (2004) and many state guidelines for identifying specific learning disabilities” (Breaux 2020, p. 113).^10^ The *Decoding* composite “provides an estimate of decontextualized phonic decoding and word reading skill” (Breaux 2020, p. 113), while *Phonological Processing* “measures phonemic proficiency and phonic decoding” (Breaux 2020, p. 114). The three composite scores are not independent, since the Pseudoword Decoding subtest is part of all three composites, while the Phonemic Proficiency and Word Reading subtests are both part of two composites. The WIAT-4 authors do not provide a justification for their rationale for having three strongly overlapping cipher skills composite scores.

The two composite scores capturing word-specific knowledge are *Orthographic Processing* and *Orthographic Processing Extended*. They both provide “an overall measure of orthographic processing, including the size of an examinee’s orthographic lexicon and the quality of orthographic representations” (Breaux 2020, p. 114).^11^ The difference between the scores is that the extended version includes one additional subtest that is only available on the Q-Interactive version of the instrument (Orthographic Choice). Both composite scores involve the Orthographic Fluency subtest as well as the Spelling subtest, the latter of which is part of the writing domain.

Since contextual reading fluency and reading comprehension are both captured by a single subtest, there are no composite scores for them. There is one composite score capturing oral language/linguistic comprehension (Oral Language), which is comprised of two subtests in the oral language domain. As we noted earlier, however, the WIAT-4 authors do not include oral language as a core area of academic achievement (Breaux 2020, p. 114).

In addition to the theory-derived composite scores, there are two atheoretical composite scores in the reading domain: Reading Fluency and Reading. *Reading Fluency* “measures overall oral reading fluency skills” (Breaux 2020, p. 113). It consists of the Oral Reading Fluency, Orthographic Fluency, and Decoding Fluency subtests, although the latter is excluded in the composite for respondents not yet in third grade. The *Reading* composite score is comprised of the Word Reading and Reading Comprehension subtests, but the WIAT-4 authors are not explicit about what the composite score is designed to measure outside of stating it “balances word-level and text-level reading skills” (Breaux 2020, p. 112). According to the simple view of reading, word recognition and language comprehension represent distinct contributions to reading comprehension, so a change in students’ reading decoding skills would not necessarily result in changing their reading comprehension. Thus, there is no reason to believe the Reading score captures an attribute with functional unity.

#### Empirical Evidence for Functional Unity of Reading Attributes

The WIAT-4 technical manual provides the same two sources of empirical evidence concerning functional unity of the behaviors comprising the reading attributes as it does OAA. The longitudinal study indicated relatively strong stability for all the composite scores, with all the correlation values greater than .90 (Breaux 2020, p. 22). This provides corroborating evidence for the hypothesis that the reading attributes represented by those scores have functional unity.

For the factor analysis, we employed the same data and data analysis procedures/programs as the OAA factor analysis except that we used promax rotation instead of bi-factor.^12^ The results are given in Table A2, Table A4 and Table A6. They indicate a messy structure for the reading subtests. The word decoding/reading subtests do not dissociate into cipher skills and word-specific knowledge, but instead all hang together along with the Oral Reading Fluency subtest. The oral language/linguistic comprehension subtests do comprise a different factor, but one with the Reading Comprehension and Math Problem Solving subtests—likely because these subtests all require significant language comprehension skills. In any case, the factor analysis does not provide strong evidence for functional unity of the attributes represented by the various reading composite scores. As such, it is difficult to make a strong argument that the composite scores have measurement validity.

### 4.2. Writing

The WIAT-4 authors created the writing subtests to be consistent with the simple view of writing and its extensions (Berninger and Winn 2006; Kim et al. 2018). In this theory, the working memory system (WM) coordinates the collective contributions of transcription skills, text generation/language skills, and self-regulation skills (i.e., executive functions) required for composition. *Transcription* involves both spelling and handwriting, while *text generation* involves the creation and organization of ideas as well as the language knowledge to transcribe the ideas into written text. All of these processes drain people’s limited WM recourses, so the more writing skills people master (i.e., develop fluency) the more WM resources can be devoted to idea generation.

The WIAT-4 provides five subtests to capture the different aspects of writing, but their availability differs by grade (see Table 4). Alphabet Writing Fluency and Spelling capture transcription, while Sentence Composition and Essay Composition capture writing quality. Sentence Writing Fluency captures text writing fluency. The two oral language subtests (Listening Comprehension and Oral Expression) are the only subtests designed to capture text generation. The subtests constitute two writing composite scores: Writing Fluency and Written Expression (see Table 5). Both scores are troublesome.

#### Empirical Evidence for Functional Unity of Writing Attributes

The *Writing Fluency* composite is comprised of the two transcription subtests, but the WIAT-4 authors do not discuss it as measuring transcription. Instead, they discuss it in term of a pragmatic purpose: capture developmental difficulties with both handwriting fluency and sentence-level text writing fluency for respondents in grades 1–4 (Breaux 2020, p. 113). Even if the WIAT-4 authors did make measurement claims about the score (i.e., represent transcription attribute), the claims would be difficult to support because of the low stability estimate for Writing Fluency is (i.e., .60; Breaux 2020, p. 23).

The WIAT-4 authors state that the *Written Expression* score “estimates overall written expression skills” (Breaux 2020, p. 112). This is neither an attribute within the simple view of writing nor an attribute the WIAT-4 authors discuss in any detail, so we have to infer its meaning based on subtest composition of the Written Expression score. The Written Expression score is comprised of Alphabet Writing Fluency, Essay Composition, Sentence Composition, and Spelling, but the particular subtests involved differ across respondent grade levels (see Table 5).

Across the entire norming sample, the stability estimate for the Written Expression score is .85 (Breaux 2020, p. 22). While this is relatively strong, there is little justification for believing the behaviors that constitute it have functional unity. Word, sentence, and text level writing build upon each other, but each level also requires unique skills. For instance, sentence-writing requires grammar knowledge not required in a spelling task, and text writing requires organizational skills not tapped by sentence-writing. As a result, writing tasks at different levels of language tend to not be highly associated with each other (Berninger et al. 1994). That was often the case in our factor analytic results (see Table A1, Table A2, Table A3, Table A4, Table A5, Table A6 and Table A7). Spelling tended to load more with the decoding-oriented subtests in the reading domain, though often presented a small cross-loading with the writing measures. Although the Sentence and Essay Composition scores often loaded together, the loadings are noticeably weaker for the 20–50-year-old group than the other age groups.

### 4.3. Mathematics

All subtests in the mathematics domain are atheoretical. They were created to capture three areas in which people have mathematical difficulties: (a) math-fact fluency (i.e., recalling basic math facts quickly); (b) computation (i.e., understanding arithmetic operations and how they relate to each other and to apply computational procedures and strategies fluently); and (c) math problem solving (i.e., applying knowledge to a problem for which the solution is not known, which is designed to enhance mathematical understanding and development).

There are two mathematics composite scores: Math Fluency and Mathematics. The *Math Fluency* composite provides “a measure of overall math fluency skills” in addition, subtraction, and multiplication (Breaux 2020, p. 113). It is comprised of between two to three Math Fluency subtests, depending on the respondents’ grade level (see Table 6). The *Mathematics* composite “estimates overall mathematics skills in the domains of math problem solving and math computation” (Breaux 2020, p. 113), and is comprised of the Numerical Operations and Math Problem Solving subtests.

#### Empirical Evidence for Functional Unity of Mathematics Attributes

Across the entire norming sample, the stability estimates for both mathematics composite scores are greater than .90 (Breaux 2020, p. 22). Our factor analysis shows the Mathematics subtests do not hang together well. Across the different age groups, the Math Problem Solving subtest hangs together more with the oral language/reading comprehension subtests than any mathematics subtest. The Numerical Operations subtest joins this factor somewhat in the 12–19-year-old norming sample, and completely joins it in the in the 20–50-year-old-sample. Consequently, it is difficult to make an argument for interpreting the Mathematics composite score, much less believe that it has measurement validity. The Math Fluency subtests do appear to hang together well across all the age groups, which corroborates the hypothesis that the math fluency attribute has functional unity. As such, the Math Fluency composite could have measurement validity.

## 5. Evaluating the Equal-Interval Claim

Earlier we stated the WIAT-4 authors make a strong claim that some score values are on an equal-interval scale. The authors define an *equal-interval scale* as meaning “that a particular size of difference [i.e., interval] between two scores represents the same amount of difference in the skill [i.e., attribute] being measured regardless of where on the scale the scores fall” (Breaux 2020, p. 64). For example, if math fluency is measured on an equal-interval scale, then a change in Math Fluency score values from, say, 90 to 110 would represent the same change in the math fluency attribute as a score value change from 60 to 80. It is not uncommon for psychological instrument authors to claim that at least some of their score values have the equal-interval property (e.g., Kaufman et al. 2014, p. 91; Wechsler et al. 2014, pp. 14, 149) because it is necessary for many of the score interpretations that psychologists currently employ. For example, in the WIAT-4 the equal-interval property is necessary for interpretive steps 2a–4 as well as the two score analysis procedures the WIAT-4 authors suggest employing for identifying respondents with a specific learning disability/disorder (Breaux 2020, pp. 83–87).

Just as common as the equal-interval claim for psychological instrument scores is the lack of support for the claim.

To some extent this is understandable. Supporting the claim requires making the case that (a) the attribute of interest is a quantity, and (b) the score values that represent the attribute’s manifestations preserve the attribute’s quantitative features. Until the mid-20th century it was largely believed that making such a case for psychological attributes was impossible (Michell 1999), and even now it is not straightforward how one goes about this (Markus and Borsboom 2013). We need not go into the detail here because the WIAT-4 authors neither provide support for their equal-interval claim, nor provide sufficient data in the technical manual for other psychologists to evaluate the claim empirically. Thus, we can only approach our evaluation of the equal-interval claim conceptually. We will do so for scores from two distinct, but typical, subtests: Numerical Operations and Math Fluency.

### 5.1. Numerical Operations

The *Numerical Operations* (NO) score “measures math computation skills” (Breaux 2020, p. 107) by capturing responses to items requiring mathematics computations ranging from naming numbers to basic calculus. By definition, if the math computation skills (MCS) attribute is a quantity, then it has the properties of equivalence, order, and additivity (Borsboom 2005; Hand 2004; Michell 1990). These are all technical concepts in measurement, but we can get by with their common-sense or intuitive meanings.

*Equivalence* roughly means that we can class any two people as either having distinguishable or indistinguishable forms of the attribute. If we can rank the distinguishable classes based on some feature of the attribute (e.g., amount, strength), then the attribute has *order*.^13^ Having order means we can rank the equivalence classes, but tells us nothing about how much one class differs from another. It is only attributes with *additivity* that it makes sense to state whether the difference between any two classes is equivalent to the difference between any two other classes. For example, if MCS has additivity, then the difference between, say, the 10th ranked class and 20th ranked class is twice as much as the difference between the 15th ranked class in the 20th ranked class.

The WIAT-4 produces multiple value units for each score, but we focus first on the raw score unit. For NO raw score values to have equal-interval property, MCS needs to be a quantity and the NO raw score values need to represent MCS faithfully. That is, the NO raw score values need to represent MCS’s equivalence, order, and additivity. If any one of these is not represented faithfully, then the NO raw score values cannot have equal intervals. We will assume MCS is a quantity and focus on NO representing its order property.

For the NO raw score values to represent the order of MCS faithfully, certain conditions must hold (Coombs et al. 1954). Specifically, the NO raw score values must be such that: (a) all respondents that are in the same MCS class (i.e., equivalent forms of MCS) have the same NO value, and all respondents that are in different MCS class (i.e., non-equivalent forms of MCS) have different NO values; (b) an order relation exists between respondents at each possible pair of NO values (e.g., respondents with a NO value of 100 have more MCS than respondents with a NO value of 99); and (c) there is consistency in the order relations (e.g., if respondents with a NO value of 100 have more MCS than those with a NO value of 99, and those with a NO value of 99 have more MCS than those with NO value of 98, then respondents with a NO value of 100 have more MCS than those with a NO value of 98). These conditions cannot be guaranteed to be true for the NO raw score values.^14^

The NO raw score is a behavior count consisting of the number of items a respondent correctly answered, and each item contributes the exact same to the raw score.^15^ The items are not exchangeable, however, because they differ in content and difficulty. These features combine to allow for situations in which two respondents have the same NO score, yet answer different sets of items correctly and, potentially, have different MCS levels. For example, there are 495 ways to have a raw score of 4 on an instrument with 12 items.^16^ Not all 495 of those patterns are possible, but if just one-fifth of them are, then that would still allow for 99 possible response patterns that produce a raw score of 4. The number of possible combinations of correct and incorrect responses expands rapidly as the number of items increases, and the NO subtest has over 50 items. Thus, it is highly probable that respondents with the same raw score have noticeably distinct response patterns. To the extent this is true, the structure of the NO raw score values is not guaranteed to represent the order of MCS faithfully. As such, the NO raw scores could not represent the additivity of MCS, and thus, cannot comprise an equal-interval scale.

To some extent it is moot whether the raw score has validity because the WIAT-4 authors strongly discourage interpreting those score values—although for reasons other than we discussed (see Breaux 2020, p. 64). As an alternative, the WIAT-4 authors suggest interpreting one of the seven other units available for each score (i.e., standard, percentile rank, normal curve equivalent, stanine, age equivalent, grade equivalent, growth scale).^17^ We will focus on the standard score unit because the WIAT-4 authors claim these values are on an equal-interval measurement scale (Breaux 2020, p. 64).

The WIAT-4 authors implicitly define a *standard score* using Equation (1) (Breaux 2020, p. 64).

(1) $$\mathrm{Standard}=\left(\frac{Raw-\bar{Raw}}{SD_{Raw}}\right)\times 15+100,$$

where *Raw* is the raw score for a particular respondent, $\bar{Raw}$ is the mean raw score in the selected norm group, and $SD_{RAW}$ is the raw score standard deviation in the norm group.^18^ An equivalent way of writing Equation (1) is in slope-intercept form, which is shown in Equation (2).

(2) $$\mathrm{Standard}=\frac{\left(100\times SD_{Raw}\right)-\left(\bar{Raw}\times 15\right)}{SD_{Raw}}+Raw\left(\frac{15}{SD_{Raw}}\right).$$

Since $\bar{Raw}$ and $SD_{RAW}$ are constants for a particular set of same-age or same-grade respondents, Equation (2) makes two things explicit. First, standard scores are just linear transformations of raw scores. As a linear transformation, the standard score conversion does not change anything about the raw score’s structure, much less the structure of the represented attribute^19^. Instead, it just alters the meaning of the score values’ origin (i.e., 0) and unit (i.e., 1). Thus, standard scores do not represent MCS any more faithfully than raw scores. If the raw scores values were not originally on an equal-interval scale, then the standard scores will not be one an equal-interval measurement scale either.

Second, standard score units are in standard deviations, so they are statistical units that represent variable dispersion within a group of respondents. They are not *measurement units*, which are particular manifestations of an attribute of interest used to represent other manifestations of the same attribute (Joint Committee for Guides in Metrology 2012). Score values expressed in standard deviations may have equal intervals on some statistical distribution, but it does not follow that the score values have equal intervals with respect to the attribute of interest. On the contrary, there is no reason to believe that changing some score unit to a standard deviation unit imbues the scores with any additional properties concerning the attribute of interest (Boring 1920).

An illustration will make this point more concrete. For kindergarten students in the fall of the academic year, average performing students (i.e., standard score of 100) can add single digits together, while students performing one standard deviation below the mean (i.e., standard score of 85) can identify numerals. The skill gap is starkly different from the same standard score difference for students in 12th grade. Average performing 12th grade students can solve algebraic equations and use geometry skills, while 12th grade students performing one standard deviation below the mean are likely struggling with fraction operations. Thus, even though the statistical unit-based scores are the exact same for both kindergarten and 12th grade students, the meaning of those scores with respect to MCS differs substantially.

### 5.2. Math Fluency

The *Math Fluency* composite provides “a measure of overall math fluency skills” (MFS) in addition, subtraction, and multiplication (Breaux 2020, p. 113). Each item in all three subtests consists of a single addition, subtraction, or multiplication problem that respondents solve correctly or incorrectly. There are two sets of items for the subtest, with the set a particular respondent receives being based on the respondent’s grade. Respondents complete as many problems as possible within 60 seconds.

Since fluency instruments are administered under strong time constraints, it is not uncommon to believe that raw scores from these instruments represent some attribute in an equal-interval unit (e.g., problems solved-correctly-per-minute). It is true that time is a base measurement unit for the physical sciences, so has equal-interval property. Nonetheless, dividing something by time does not necessarily put the resulting values in a base measurement unit (Boring 1920; Thomas 1942). This is because instruments designed to assess the speed of something and instruments designed capture speeded procedures are two different classes of instruments (Guttman and Levy 1991).

Instruments designed to assess speed are employed when time is part of the attribute’s meaning. In psychology, the attribute is typically *response latency*, which is the time between the presentation of a stimulus (i.e., item) and the response. For example, if we are interested in measuring math fact retrieval speed, then we would present a math problem (e.g., “2 + 7 = ?”) and immediately begin some a timing device that we would stop once the respondent provides the answer. Since scoring involves capturing the time it takes to respond rather than correctness, these instruments only contain items to which respondents are expected to answer correctly.

Instruments designed to capture speeded procedures consist of completing a set of items under strong time constraints. Typically, the constraints are so strong that respondents are not expected attempt all the items, and the non-response items are coded as being incorrect. Thus, responses are scored based on a correctness criterion rather than the time it takes to respond to any given item. This makes the raw score a count of the items correctly answered within in a certain period of time, which does not necessarily entail the values have an equal-interval unit (but see Johnson et al. 2019). The Math Fluency subtests belong to this class of instruments rather than the latency class. Thus, respondents who progress from, say, answering 50 problems per minute to answering 80 problems per minute do not necessarily have the same increase in MFS as respondents who progress from answering 270 problems per minute to answering 300 problems per minute—even though both changes involve 30 problems per minute.

To some extent, Math Fluency raw scores are irrelevant because the WIAT-4 authors provide no guidance for interpreting the values. Instead, they strongly suggest interpreting standard scores. As with the Numerical Operations subtest, however, transforming Math Fluency raw scores to standard scores does not give the score values additional properties with respect to representing the attribute of interest. Thus, if the Math Fluency raw score values do not have equal intervals, then there is no reason to believe that the Math Fluency standard score values will have equal intervals either.

## 6. Conclusions

The WIAT-4 is the latest iteration of a popular instrument designed to assess academic achievement in people across a wide variety of ages and grades. The WIAT-4 authors make two strong claims about the instrument: (a) the scores can be used for measurement purposes; and (b) some of the scores (i.e., standard scores) have values with equal intervals (Breaux 2020, pp. 1, 28, 64). Before psychologists adopt an instrument and interpret the scores in a manner consistent with the authors’ claims, however, there should be sufficient evidence to support the claims (American Educational Research Association et al. 2014; International Test Commission 2001).

In this article, we evaluated the WIAT-4 authors’ measurement claims (i.e., validity evidence) for the instrument’s composite scores. Based only on the information provided in the WIAT-4 technical manual, we found the WIAT-4 authors did not provide sufficient evidence to support their measurement claims for the composite scores. First, many of the attribute concepts the scores ostensibly represent are ill defined in the technical manual (e.g., overall academic achievement) and it is unclear what attribute some of the scores are supposed to represent (e.g., Reading). As such, these scores’ values cannot be measurement values. Second, even for some of the attribute concepts with more clear meaning (e.g., cipher skills), the subtests that comprise the composite scores do not hang together in expected ways (i.e., do not appear to have functional unity). This makes it doubtful that the scores’ values are measurement values.

There are a few attribute concepts the WIAT-4 authors discuss that have the potential for measurement (e.g., math fluency skills). For the scores to have measurement validity, however, the known properties of the attribute’s manifestations need to be represented by the score values (Michell 1990; Joint Committee for Guides in Metrology 2012). Since the WIAT-4 authors claim that instrument’s standard scores are on an equal-interval scale, this entails that (a) the attributes are quantities (i.e., manifestations have equivalence, order, and additivity); and (b) the relations among standard score values faithfully represent these relations among the attribute manifestations. The WIAT-4 authors provide no evidence in the technical manual to support their equal-interval claims, and our *prima facie* analysis of the claims was not favorable. As such, we highly doubt that the scores equal-interval properties. Thus, if the attributes the WIAT-4 capture are really quantities, then the scores that represent them are not doing so validity.

### Practical Implications

The major practical implication of our evaluation concerns the appropriateness of the WIAT-4 authors’ score interpretation guidance (Breaux 2020, pp. 77–79). First, step 1 in the interpretive guidance should be removed because the Total Achievement score should not be interpreted clinically. The score is supposed to represent overall academic achievement (OAA), but it is doubtful that OAA is even a clinically useful attribute concept, much less a unitary attribute. Based on the information provided in the technical manual, we cannot state that the Total Achievement score is anything more than a sum of items unique to the WIAT-4 instrument that the WIAT-4 authors believe are important.

Second, some of the composite scores may be useful for ranking students (step 2a in the interpretive guidance), but the evidence in the technical manual is insufficient to support the practice of interpreting quantitative differences in the composite scores (steps 2b–3) or subtest scores (step 4). Thus, any score comparisons should be limited to qualitative differences.

For example, pretend Zsa Zsa has an age-based Math Fluency standard score of 85 and a Reading Fluency standard score of 115. From this information, we can state her ability to conduct basic mathematics operations quickly is currently lower than the average ability of her same-age peers in the United States, while her oral reading ability for relatively simple English words is currently higher than her peers’ average. It would be incorrect to interpret the 115–85 = 30-point difference between the scores because that the meaning of the 30-point difference differs across the score distributions. That is, even though numerically 115–85 = 90–60 = 130–100 = …, the meanings of the score differences with respect the represented attributes are not equivalent. Even qualitative interpretations of the differences in standard scores need to be done cautiously (Woodcock 1999). That is, interpreting the scores as indicating that Zsa Zsa’s oral reading ability is “more developed” than her mathematics operations ability would not be warranted unless we had additional evidence (e.g., homework, motivation level; Shapiro 2011).

Although our evaluation is not supportive of the WIAT-4 authors’ measurement validity claims, our evaluation is agnostic regarding whether psychologists should employ the instrument’s scores for other purposes. Psychologists have a long history of employing instruments that produce scores that have utility (i.e., aid in making decisions) without measuring any attribute (e.g., Binet-Simon, Minnesota Multiphasic Personality Inventory; Berg 1959). Given the WIAT-4 authors’ commendable revision of many scores in the reading and writing domains to align with strong theories in those areas, it is possible that those scores have utility for making decisions about respondents’ academic achievement in those areas. The WIAT-4 authors do not provide the necessary information in the technical manual to evaluate utility, however, so it will remain for future evaluations to determine whether WIAT-4 users should employ the scores for decision-making purposes.

On a final note, some readers of this article may believe that our evaluation of the WIAT-4 is out-of-sync with how psychologists currently think about validity and evaluate the validity of psychological instruments (e.g., Messick 1989). We acknowledge that the framework in which we evaluated the WIAT-4 is different from the received view of validity that permeates documents such as the American joint test standards (American Educational Research Association et al. 2014) or the European Federation of Psychologists’ Associations model for instrument evaluation (Evers et al. 2013). We also acknowledge that the received view has been criticized extensively (e.g., Barrett 2018; Markus and Borsboom 2013). This criticism is not recent, however, but has a relatively long history in psychology. More than 40 years ago, Oscar Buros (1977) wrote, “If we make it our goal to measure rather than to differentiate, most of our methods of constructing tests, measuring repeatability, assessing validity, and interpreting test results will need to be drastically changed” (p. 12). It is our belief that our evaluation is fully in line with this needed drastic change.

## Figures and Tables

**Table 1:** Wechsler Individual Achievement Test—Fourth Edition Subtests.

Subtest Scores	Grade Levels	Composite Scores	
		Single Knowledge Domain	Multiple Knowledge Domains
Reading Domain			
Decoding Fluency ^a^	3–12+	Reading Fluency (3–12+)	
Oral Reading Fluency	1–12+	Reading Fluency (1–12+)	
Orthographic Fluency ^a^	1–12+	Dyslexia Index (4–12+)	Orthographic Processing (1–12+)
		Reading Fluency (1–12+)	
Phonemic Proficiency ^a,b^	PK–12+	Basic Reading	
		Dyslexia Index (PK-3)	
		Phonological Processing (1–12+)	
Pseudoword Decoding	1–12+	Basic Reading	
		Decoding	
		Dyslexia Index (4–12+)	
		Phonological Processing (1–12+)	
Reading Comprehension	K-12+	Reading (K-12+)	Total Achievement (PK-12+)
Word Reading	PK-12+	Basic Reading	Total Achievement (PK-12+)
		Decoding	
		Dyslexia Index (PK-12+)	
		Reading (K-12+)	
Writing Domain			
Alphabet Writing Fluency	PK-4+	Written Expression (K-1)	Total Achievement (PK-1)
		Writing Fluency (1-4)	
Essay Composition	3-12+	Written Expression (4-12+)	Total Achievement (4–12+)
Sentence Composition ^c^	1-12+	Written Expression (2-12+)	Total Achievement (2–3)
Sentence Writing Fluency ^a^	1-12+	Writing Fluency (1-4)	
Spelling	K-12+	Written Expression (K-12+)	Total Achievement (K-12+)
			Orthographic Processing (1–12+)
Mathematics Domain			
Math Problem Solving	PK-12+	Mathematics (K-12+)	Total Achievement (PK-12+)
Numerical Operations	K-12+	Mathematics (K-12+)	Total Achievement (K-12+)
Math Fluency–Addition	1–12+	Math Fluency (1–12+)	
Math Fluency–Subtraction	1–12+	Math Fluency (1–12+)	
Math Fluency-Multiplication	3–12+	Math Fluency (3–12+)	
Oral Language Domain			
Listening Comprehension ^d^	PK-12+	Oral Language (PK-12+)	
Oral Expression ^e^	PK-12+	Oral Language (PK-12+)	

Note. There is an additional new subtest called *Orthographic Choice*, but it is only available on the Q-Interactive version of the instrument. It combines with the Orthographic Fluency and Spelling subtests to form an Orthographic Processing Extended composite score. ^a^ Subtest is new to WIAT-IV. ^b^ Listed as a subtest in the Language Processing domain in technical manual. ^c^ Listed as a subtest in the technical manual but is comprised of two “component scores:” Sentence Building and Sentence Combining. ^d^ Listed as a subtest in the technical manual but is comprised of two “component scores:” Receptive Vocabulary and Oral Discourse Comprehension ^e^. Listed as a subtest in the technical manual but is comprised of three “component scores:” Expressive Vocabulary, Oral Word Fluency, and Sentence Repetition.

**Table 2:** Indices informing on number of factors to extract.

Number of Factors	χ^2^	*df*	CFI	RMSEA (95% CI)	AIC	BIC	Eigen > 1	Parallel Analysis	MAP
4–7 Years									
5	604.055	61	.967	0.14 (0.13–0.15)	482.055	227.454	3	5	3
4	837.031	74	.954	0.15 (0.14–0.16)	689.031	380.170			
3	1080.827	88	.940	0.15 (0.15–0.16)	904.827	537.533			
8–11 Years									
5	284.022	100	.991	0.06 (0.05–0.07)	84.022	−333.356	4	5	3
4	468.924	116	.982	0.08 (0.07–0.09)	236.924	−247.236			
3	747.181	133	.969	0.10 (0.09–0.10)	481.181	−73.933			
12–19 years									
5	185.215	86	.995	0.05 (0.04–0.06)	13.215	−345.731	3	5	3
4	279.639	101	.991	0.06 (0.05–0.07)	77.639	−343.914			
3	412.250	117	.986	0.07 (0.06–0.08)	178.250	−310.083			
20–50 years									
5	458.797	86	.978	0.10 (0.09–0.10)	286.797	−72.148	4	3	3
4	594.135	101	.971	0.10 (0.09–0.11)	392.135	−29.417			
3	803.818	117	.960	0.11 (0.10–0.12)	569.818	81.485			

Note. AIC: Akaike information criterion; BIC: Bayesian information criterion, RMSEA: root mean square error of approximation, CFI: comparative fit index, MAP: Minimum average partial. χ^2^ and χ^2^-based fit indices (CFI, RMSEA, AIC, and BIC) were estimated used maximum likelihood extraction.

**Table 3:** WIAT-4 Subtest and Composite Scores Aligned with the Simple View of Reading and its Extensions.

Reading Component	WIAT-4	
	Subtests	Composites
Word Decoding/Reading: Cipher Skills	Decoding Fluency	Basic Reading
	Phonemic Proficiency	Phonological Processing
	Pseudoword Decoding	Decoding
	Word Reading	
Word Decoding/Reading: Word-Specific Knowledge	Orthographic Choice ^a^	Orthographic Processing
	Orthographic Fluency	Orthographic Processing Extended ^a^
	Spelling ^b^	
Oral Language/Linguistic Comprehension	Oral Language ^c^	Listening Comprehension ^c^
	Oral Expression ^c^	Oral Expression ^c^
Contextual Reading Fluency		Oral Reading Fluency
Reading Comprehension		Reading Comprehension

Adapted from Breaux (2020, p. 91). ^a^ Only available on Q-Interactive version of the instrument. ^b^ Part of writing domain. ^c^ Part of oral language domain.

**Table 4:** WIAT-4 Subtest Aligned with the Simple View of Writing and its Extensions.

Writing Component	Grades	WIAT-4 Subtests
Transcription	PK-4	Alphabet Writing Fluency
	K-12+	Spelling
Text Generation	PK-12+	Listening Comprehension ^a^
	PK-12+	Oral Expression ^a^
Text Writing Fluency	1–12+	Sentence Writing Fluency
Writing Quality	1–12	Sentence Composition
	3–12+	Essay Composition

Adapted from Breaux (2020, p. 95). ^a^ Part of oral language domain.

**Table 5:** WIAT-4 Writing Composite Scores.

Composite Score	Grades	Subtests
Writing Fluency	1–4	Alphabet Writing Fluency & Spelling
Written Expression	K–1	Spelling & Alphabet Writing Fluency
	2–3	Spelling & Sentence Composition
	4–12	Spelling, Sentence Composition, & Essay Composition

Adapted from Breaux (2020, pp. 112–13).

**Table 6:** WIAT-4 Mathematics Scores.

Area of Mathematics Difficulty	WIAT-4	
	Subtest Scores	Composite Scores
Math-fact fluency	Math Fluency–Addition	Math Fluency
	Math Fluency–Subtraction	
	Math Fluency–Multiplication ^a^	
Computation	Numerical Operations	Mathematics
Math problem solving	Math Problem Solving	Mathematics

Adapted from Breaux (2020, pp. 112–13). ^a^ Available only for respondents in grade 3 or higher.

## Data Availability

Data from this study came from the WIAT-4 Technical Manual.

## Notes

1 For convenience, we use the term *authors* throughout the article instead of the more accurate term *construction agency*. Nearly all modern standardized instruments are created by a team of people with different specialty knowledge (e.g., content matter, test construction techniques, item analysis), only a portion of which are credited on instrument documentation.

2 We consider behavior to be a subclass of doings (Maraun 2013).

3 Functional unity is applicable to phenomena from a variety of disciplines and knowledge domains, so may involve things other than behavior (e.g., neural activity).

4 Technically, we *classify* attributes represented on a nominal scale rather than measure them. Classification has some properties similar to measurement, but they are distinct processes.

5 The WIAT-4 authors provide some utility evidence for the Dyslexia Index score, but do not describe how they gathered this evidence in any detail (Breaux 2020, p. 114).

6 Steps 2a and 2b are combined into a single Step 2 in the technical manual.

7 Idiosyncratic employments of the intelligence concept continued throughout the 20th century and continue today (Legg and Hutter 2007).

8 The bi-factor rotation requires extracting *p* + 1 factors, with the *p* indicating the number of group factors and *+1* indicating the additional general factor. Thus, we actually extracted 4–6 factors.

9 We use the term *triadic theory* instead of the more common *gf –gc theory.* The latter term once had a specific meaning, but now it is more ambiguous as it can refer to either the theory Raymond Cattell created to extend Spearman’s nöegenetic theory or the refinements and expansions to *gf –gc* theory initiated by Cattell’s student, John Horn. Although Horn and Cattell worked together occasionally throughout Cattell’s life, by the 1970s they had independent research programs and had developed separate intelligence theories. Thus, except for historical purposes, *gf –gc* theory is no longer viable because it has been replaced with two competing theories: Horn’s *extended Gf–Gc theory* and Cattell’s *triadic theory*.

10 The acronym IDEA stands for the Individuals with Disabilities Education Act, which is an American law passed in 2004.

11 The term *orthographic lexicon* is a more technical term for sight vocabulary (i.e., words we can correctly read instantly without effort).

12 Promax rotation is oblique, meaning it allows the factors to be correlated.

13 There are other ways for the classes to be ordered, but since we are employing common sense/intuitive meanings, we will not differentiate among them (for more details, see Michell 1999).

14 Measurement models guaranteeing the conditions are not necessary, but the WIAT-4 authors do not discuss alternative probabilistic models in the technical manual.

15 Some WIAT-4 subtests offer partial credit, so the raw scores would be the number of points earned.

16 There are 495 ways to combine 4 out of 12 objects (i.e., _12_*C*_4_).

17 The WIAT-4 provides both age- and grade-based norm groups for the norm-referenced scores, so it is likely more accurate to state the WIAT-4 provides 11 different score units in addition to the raw score.

18 The WIAT-4 standard scores are all integers, so the values from Equation (1) must be rounded. The WIAT-4 authors do not provide information about the rounding function they employ, however, so we do not include one in Equation (1).

19 The WIAT-4 authors hint, but do not state explicitly, that they normalized the raw score values within a norm group before converting to standard scores. It is true that normalizing can make a score’s values have certain statistical properties, but it does not follow that the attribute the score values represent gains properties because of normalizing (Michell 2020; Thomas 1982). Thus, normalizing the raw scores does not change our evaluation that the WIAT-4 authors do not provide sufficient support for their claim that standard scores are on an equal-interval measurement scale.

## Appendix A

**Table A1:** Oblique Factor Correlations Across Age Groups and Factor Models.

	Age 4 to 7					Age 8 to 11					Age 12 to 19					Age 20 to 50				
Five Factor Models																				
	F1	F2	F3	F4	F5	F1	F2	F3	F4	F5	F1	F2	F3	F4	F5	F1	F2	F3	F4	F5
Factor 1	1.00					1.00					1.00					1.00				
Factor 2	.66	1.00				.66	1.00				.72	1.00				.35	1.00			
Factor 3	.69	.57	1.00			.52	.58	1.00			.61	.62	1.00			.60	.13	1.00		
Factor 4	.73	.67	.63	1.00		.33	.40	.40	1.00		.64	.41	.41	1.00		.63	.45	.59	1.00	
Factor 5	.46	.36	.41	.50	1.00	.60	.62	.50	.29	1.00	.74	.76	.67	.44	1.00	.46	.34	.23	.58	1.00
SS Loadings ^1^	4.85	2.39	1.45	1.43	1.16	4.94	2.70	3.09	.85	1.65	3.63	2.75	3.10	1.46	1.61	5.63	2.41	1.01	2.44	.88
Prop Tot Variance ^1^	.29	.14	.09	.08	.07	.25	.13	.15	.04	.08	.19	.14	.16	.08	.08	.30	.13	.05	.13	.04
Prop Comm Var ^1^	.43	.21	.13	.13	.10	.37	.20	.23	.06	.12	.29	.22	.25	.12	.13	.45	.20	.08	.20	.07
Four Factor Models																				
	F1	F2	F3	F4		F1	F2	F3	F4		F1	F2	F3	F4		F1	F2	F3	F4	
Factor 1	1.00					1.00					1.00					1.00				
Factor 2	.70	1.00				.65	1.00				.65	1.00				.37	1.00			
Factor 3	.58	.51	1.00			.51	.57	1.00			.56	.59	1.00			.63	.51	1.00		
Factor 4	.72	.65	.50	1.00		.61	.61	.52	1.00		.70	.77	.65	1.00		.55	.08	.40	1.00	
SS Loadings ^1^	4.88	2.74	1.88	1.30		4.97	2.79	3.17	1.69		4.37	2.53	3.01	2.26		5.54	3.04	2.57	.82	
Prop Tot Variance ^1^	.29	.16	.11	.08		.25	.14	.16	.08		.23	.13	.16	.12		.29	.16	.14	.04	
Prop Comm Var ^1^	.45	.25	.17	.12		.39	.22	.25	.13		.36	.21	.25	.19		.46	.25	.21	.07	
Three Factor Models																				
	F1	F2	F3			F1	F2	F3			F1	F2	F3			F1	F2	F3		
Factor 1	1.00					1.00					1.00					1.00				
Factor 2	.71	1.00				.70	1.00				.60	1.00				.41	1.00			
Factor 3	.67	.61	1.00			.55	.58	1.00			.72	.64	1.00			.63	.55	1.00		
SS Loadings ^1^	5.08	2.88	2.40			5.59	3.01	3.30			4.87	3.77	3.10			5.53	3.11	2.77		
Prop Tot Variance ^1^	.30	.17	.14			.28	.15	.17			.26	.20	.16			.29	.16	.15		
Prop Comm Var ^1^	.49	.28	.23			.47	.25	.28			.41	.32	.26			.48	.27	.24		

Note. Variance proportions may not appear in descending order. They were re-ordered for comparison to the tables of factor loadings; ^1^—For oblique rotations, we report variance accounted via the rotated factor loadings, accounting for correlations between factors.

**Table A2:** Five Factor Oblique Solution Across Age Groups.

	Factor 1				Factor 2				Factor 3				Factor 4				Factor 5				Communalities ^1^			
	4–7	8–11	12–19	20–50	4–7	8–11	12–19	20–50	4–7	8–11	12–19	20–50	4–7	8–11	12–19	20–50	4–7	8–11	12–19	20–50	4–7	8–11	12–19	20–50
WR	**.98**	**.77**	**.76**	**.99**	−.04	.21	.24	.09	.14	−.13	−.09	−.14	−.06	−.12	.09	−.12	−.12	.14	−.08	−.06	.92	.83	.80	.79
RC	**.36**	.14	.02	.24	**.36**	**.67**	**.62**	**.41**	.10	−.08	−.05	.28	−.07	.09	.07	−.10	.08	.03	.13	−.02	.58	.61	.55	.55
PD	**.72**	**.86**	**1.07**	**.87**	.15	−.02	−.17	−.01	−.01	−.05	.03	−.04	−.09	−.06	−.03	−.04	.10	.07	−.08	.07	.73	.71	.82	.81
OF	**.99**	**.76**	.23	**.79**	.01	.19	.26	−.11	−.14	.08	.05	.11	.05	.04	**.55**	−.02	.02	−.14	.01	.10	.89	.77	.83	.83
DF	-	**1.02**	**.60**	**1.05**	-	−.13	−.10	−.19	-	.10	.11	-.08	-	.05	**.44**	.13	-	−.15	−.09	−.19	-	.86	.80	.81
ORF	**.94**	**.68**	.12	**.62**	−.01	.11	−.01	.10	−.29	.15	.07	.19	.17	.08	**.53**	−.02	−.04	−.16	.19	−.09	.65	.63	.59	.60
SP	**.56**	**.68**	**.54**	**.85**	−.23	−.10	.01	.01	.27	−.02	−.01	−.14	−.02	−.01	.12	.03	**.30**	**.41**	**.32**	.21	.74	.81	.77	.77
SC	.26	−.04	.13	.13	**.38**	.28	.12	.06	−.04	.03	−.05	.10	−.05	.14	−.05	−.06	.28	**.57**	**.60**	**.53**	.57	.68	.56	.56
EC	-	.17	−.07	−.13	-	−.25	−.12	.04	-	.05	-.02	.29	-	.06	.12	.04	-	**.75**	**.80**	**.51**	-	.57	.49	.50
AWF	.26	−.05	-	-	−.16	.01	-	-	**.32**	−.02	-	-	.17	**.75**	-	-	**.33**	.17	-	-	.31	.63	-	-
SWF	−.05	.03	−.05	−.08	.05	.19	.16	−.24	−.09	.23	.26	**.50**	.15	.30	.22	.18	**.74**	−.11	−.04	.13	.51	.30	.20	.20
MPS	−.11	−.02	.15	−.02	**.49**	**.59**	**.57**	**.91**	**.43**	**.36**	**.33**	−.20	.14	−.19	−.16	.18	−.15	.10	.02	.01	.69	.71	.77	.77
NO	.01	−.06	−.05	−.09	.00	.19	**.31**	**.92**	**.82**	**.59**	**.43**	**−.31**	.08	−.02	−.13	.21	−.04	.24	.29	.03	.63	.68	.68	.68
MFA	−.06	−.01	.05	.07	−.03	−.07	−.09	.11	.04	**.88**	**.96**	.18	**.76**	−.01	.05	**.81**	.17	−.02	−.10	−.12	.79	.68	.81	.80
MFS	.07	−.02	.02	.00	.12	−.01	.01	−.06	.12	**.94**	**.93**	**.30**	**.66**	−.05	.05	**.83**	.05	.01	−.08	.10	.75	.83	.84	.85
MFM	.09	.08	−.05	−.03	-	−.14	−.05	.10	-	**.84**	**.85**	-.02	-	.09	.07	**.82**	-	.01	.11	.00		.73	.80	.80
LC	−.05	.06	−.07	.16	**.89**	**.91**	**1.00**	**.32**	−.08	−.09	−.07	**.38**	.03	−.06	.08	−.08	−.11	−.21	−.21	.13	.58	.60	.64	.66
OE	−.03	−.02	.01	**.33**	**.79**	**.76**	**.67**	**.35**	−.06	−.02	−.04	.18	.02	.13	.10	−.08	.12	−.03	.09	.04	.66	.60	.59	.60
PP	.26	**.46**	**.68**	**.48**	**.34**	.18	.17	.12	.22	−.09	−.02	.26	−.13	.04	−.12	.11	.16	.21	−.01	−.06	.63	.50	.53	.56
OC	**.81**	**.58**	**.35**	**.64**	−.01	−.04	.03	.09	.13	−.02	.01	−.14	.09	−.06	.09	.16	−.23	.26	**.31**	.12	.65	.51	.51	.73

Note. Factor loadings greater than 1 suggest Heywood cases. ^1^—Communalities represent the final estimates from the unrotated solution; WR—Word Reading; RC—Reading Comprehension; PD—Pseudoword Decoding; OF—Orthographic Fluency; DF—Decoding Fluency; ORF—Oral Reading Fluency; SP—Spelling; SC—Sentence Composition; EC—Essay Composition; AWF—Alphabet Writing Fluency; SWF—Sentence Writing Fluency; MPS—Math Problem Solving; NO—Numerical Operations; MFA—Math Fluency Addition; MFS—Math Fluency Subtraction; MFM—Math Fluency Multiplication; LC—Listening Comprehension; OE—Oral Expression; PP—Phonemic Proficiency; OC—Orthographic Choice; Columns represent age groups, 4 to 7, 8 to 11, 12 to 19, 20 to 50. **Bold** coefficients are greater than .30.

**Table A3:** Bifactor Solution with Five Specific Factors Across Age Groups.

	General Factor				Factor 1				Factor 2				Factor 3				Factor 4				Factor 5				Communalities			
	4–7	8–11	12–19	20–50	4–7	8–11	12–19	20–50	4–7	8–11	12–19	20–50	4–7	8–11	12–19	20–50	4–7	8–11	12–19	20–50	4–7	8–11	12–19	20–50	4–7	8–11	12–19	20–50
WR	**.90**	**.85**	**−.87**	**.88**	−.25	−.09	−.05	.03	−.07	.01	−.03	.02	−.01	−.14	.13	−.13	.08	−.23	.05	−.04	.14	.12	.14	.14	.91	.82	.79	.81
RC	**.72**	**.69**	**−.66**	**.64**	−.07	.07	.01	.03	.22	**.34**	**.34**	**−.37**	.03	.00	.00	.01	.07	−.06	−.01	.22	−.03	−.12	−.03	−.04	.58	.61	.55	.59
PD	**.87**	**.72**	**−.84**	**.87**	−.01	−.14	.02	.18	.00	−.05	−.29	.00	−.25	−.04	.09	−.07	.06	**−.58**	−.03	−.08	.10	.05	.11	.01	.83	.88	.81	.80
OF	**.85**	**.84**	**−.83**	**.83**	**−.55**	**−.35**	**−.38**	**−.35**	.00	.02	.03	.03	.00	.01	−.02	−.07	.03	−.01	.03	−.07	−.05	.02	.03	−.01	1.02	.83	.83	.81
DF	-	**.77**	**−.80**	**.81**	-	**−.38**	**−.34**	−.03	-	−.11	−.24	.06	-	.03	.00	.03	-	**−.30**	−.03	−.26	-	.00	.07	.22	-	.84	.81	.77
ORF	**.67**	**.75**	**−.66**	**.73**	**−.43**	**−.36**	**−.38**	**−.31**	−.03	−.01	−.07	−.19	−.10	.06	−.05	−.02	−.09	.02	.02	.01	.03	−.02	−.09	.12	.65	.69	.60	.68
SP	**.84**	**.86**	**−.86**	**.90**	.00	−.01	−.04	.02	−.22	−.22	−.05	.13	−.02	−.05	.02	.01	−.01	−.15	.18	.03	−.06	.04	−.07	−.04	.76	.81	.77	.83
SC	**.72**	**.72**	**−.67**	**.50**	.05	**.34**	.11	−.06	.20	.03	.13	.06	−.13	.08	−.02	−.01	.03	.01	.02	.24	−.10	−.15	−.29	**−.35**	.58	.66	.56	.43
EC	-	**.63**	**−.56**	**.38**	-	**.31**	−.01	.06	-	**−.38**	.04	−.08	-	.00	−.06	.09	-	.07	.05	.20	-	−.03	**−.43**	**−.45**	-	.65	.50	.41
AWF	**.44**	**.38**	-	-	−.01	.05	-	-	−.10	−.01	-	-	.25	.11	-	-	−.13	.02	-	-	−.25	**−.72**	-	-	.34	.68	-	-
SWF	**.51**	**.37**	**−.38**	.19	.00	−.08	−.15	−.21	.01	.13	.06	−.23	−.02	.23	−.19	.13	−.14	.04	−.06	−.10	**−.58**	−.27	.00	−.19	.61	.29	.20	.20
MPS	**.68**	**.70**	**−.76**	**.56**	.01	.15	.18	−.02	**.36**	.29	**.30**	−.03	**.31**	**.30**	−.25	**.39**	−.09	.02	−.07	**.60**	.05	.11	.01	.01	.70	.70	.77	.82
NO	**.64**	**.65**	**−.67**	**.45**	.11	.16	.16	.06	.03	.06	.24	.05	**.45**	**.48**	**−.35**	**.42**	−.07	.03	.02	**.60**	.03	−.03	−.14	.00	.63	.68	.68	.75
MFA	**.60**	**.44**	**−.59**	**.51**	.01	−.02	−.03	.04	−.03	.00	−.06	−.09	.05	**.70**	**−.67**	**.76**	**−.77**	−.03	−.02	.00	−.04	−.02	.01	.02	.95	.69	.80	.84
MFS	**.64**	**.53**	**−.64**	**.53**	−.03	.01	−.02	.01	.07	.02	.01	−.06	.15	**.74**	**−.67**	**.75**	**−.50**	−.02	.02	−.02	−.02	.00	.03	−.15	.69	.84	.85	.86
MFM	-	**.53**	**−.63**	**.34**	-	−.08	−.02	−.08	-	−.08	.01	.14	-	**.65**	**−.63**	**.78**	-	.07	.02	.07	-	−.08	−.08	−.01	-	.73	.80	.75
LC	**.47**	**.58**	**−.59**	**.63**	.02	−.02	−.01	−.07	**.59**	**.50**	**.53**	**−.34**	.01	−.04	.03	.02	.00	.07	.02	.23	.02	.01	.19	−.15	.58	.60	.66	.59
OE	**.62**	**.63**	**−.68**	**.67**	.03	.06	−.01	−.10	**.52**	**.41**	**.35**	−.22	.00	.05	.01	.02	.01	.09	−.10	.22	−.11	−.15	−.03	−.03	.66	.60	.60	.55
PP	**.77**	**.65**	**−.72**	**.73**	.10	.09	.13	.13	.18	.07	−.04	**−.30**	−.02	−.03	.08	.12	.10	**−.40**	−.12	−.02	−.01	−.08	.04	.01	.64	.60	.56	.66
OC	**.74**	**.68**	**−.70**	**.74**	−.24	−.05	.00	.00	−.03	−.15	.00	.13	.03	−.07	−.01	.15	−.05	−.09	**.49**	.06	.19	.09	−.04	.00	.64	.51	.73	.59
ω/ω.h	.95	.89	.92	.85	.01	.00	.00	.00	.02	.00	.01	.01	.00	.05	.04	.07	.01	.01	.00	.02	.00	.01	.00	.01				
H	.96	.95	.96	.96	.45	.44	.37	.28	.56	.51	.50	.38	.35	.77	.72	.82	.65	.48	.28	.60	.40	.56	.30	.36				
SS Loadings	8.31	8.83	9.28	8.13	.64	.71	.52	.35	.94	.89	.84	.56	.48	1.89	1.54	2.16	.92	.70	.32	1.07	.50	.70	.38	.49				
Prop Tot Variance	.49	.44	.49	.43	.04	.04	.03	.02	.06	.04	.04	.03	.03	.09	.08	.11	.05	.03	.02	.06	.03	.04	.02	.03				
Prop Comm Var	.71	.64	.72	.64	.05	.05	.04	.03	.08	.06	.07	.04	.04	.14	.12	.17	.08	.05	.02	.08	.04	.05	.03	.04				

Note. WR—Word Reading; RC—Reading Comprehension; PD—Pseudoword Decoding; OF—Orthographic Fluency; DF—Decoding Fluency; ORF—Oral Reading Fluency; SP—Spelling; SC—Sentence Composition; EC—Essay Composition; AWF—Alphabet Writing Fluency; SWF—Sentence Writing Fluency; MPS—Math Problem Solving; NO—Numerical Operations; MFA—Math Fluency Addition; MFS—Math Fluency Subtraction; MFM—Math Fluency Multiplication; LC—Listening Comprehension; OE—Oral Expression; PP—Phonemic Proficiency; OC—Orthographic Choice; Columns represent age groups, 4 to 7, 8 to 11, 12 to 19, 20 to 50. **Bold** coefficients are greater than .30.

**Table A4:** Four Factor Oblique Solution Across Age Groups.

	Factor 1				Factor 2				Factor 3				Factor 4				Communalities ^1^			
	4–7	8–11	12–19	20–50	4–7	8–11	12–19	20–50	4–7	8–11	12–19	20–50	4–7	8–11	12–19	20–50	4–7	8–11	12–19	20–50
WR	**1.03**	**.80**	**.68**	**.98**	.01	.16	.23	.04	−.03	−.16	−.12	−.12	−.09	.11	.13	−.13	.92	.82	.77	.81
RC	**.36**	.14	.08	.21	**.41**	**.69**	**.59**	**.46**	−.05	−.06	−.03	−.09	.10	.05	.14	.25	.58	.62	.55	.55
PD	**.71**	**.88**	**.76**	**.87**	.15	−.04	−.11	.06	−.11	−.06	−.03	−.05	.12	.04	.23	−.06	.73	.72	.71	.74
OF	**.96**	**.76**	**.76**	**.78**	−.04	.20	.23	.02	.01	.10	.10	−.03	.00	−.14	−.14	.09	.87	.77	.78	.69
DF	-	**1.00**	**.98**	**1.04**	-	−.10	−.08	−.35	-	.13	.12	.15	-	−.15	−.15	−.07	-	.84	.82	.78
ORF	**.84**	**.66**	**.66**	**.59**	−.11	.14	−.01	.08	.07	.18	.13	.00	−.07	−.14	−.02	.19	.59	.62	.52	.58
SP	**.60**	**.69**	**.54**	**.85**	−.17	−.10	−.03	.17	.08	−.02	−.02	.01	**.39**	**.39**	**.45**	−.14	.73	.81	.78	.80
SC	.22	−.06	.05	.16	**.37**	**.33**	.06	**.48**	−.07	.03	−.04	−.09	**.30**	**.62**	**.69**	.03	.57	.70	.56	.32
EC	-	.17	.10	−.08	-	−.23	−.12	**.47**	-	.04	.05	.01	-	**.76**	**.61**	.16	-	.58	.40	.26
AWF	−.01	−.08	-	-	−.10	.24	-	-	**.31**	.16	-	-	**.39**	.22	-	-	.29	.22	-	-
SWF	−.15	−.01	.20	−.10	−.01	.29	.15	-.03	.17	**.30**	.28	.17	**.68**	−.04	−.14	**.43**	.45	.24	.20	.17
MPS	.08	.05	−.08	−.02	**.65**	**.48**	**.52**	**.81**	.28	**.31**	.28	.21	−.11	.07	.23	−.15	.66	.62	.75	.78
NO	.13	−.04	−.19	−.09	.24	.18	.26	**.83**	**.32**	**.57**	**.40**	.23	.09	.23	**.41**	−.27	.43	.67	.68	.76
MFA	.02	−.01	.09	.06	−.08	−.07	-.08	.00	**.80**	**.88**	**.92**	**.85**	.16	−.03	−.05	.18	.74	.68	.80	.83
MFS	.06	−.01	.06	.01	.10	−.02	.01	.03	**.75**	**.92**	**.90**	**.84**	.04	.00	−.03	.25	.76	.82	.84	.85
MFM	-	.07	.02	−.02	-	−.10	−.06	.02	-	**.86**	**.84**	**.85**	-	.02	.12	−.02	-	.73	.80	.73
LC	−.10	.08	.01	.12	**.91**	**.85**	**.97**	**.51**	−.02	−.09	−.06	−.09	−.15	−.20	−.20	**.34**	.56	.57	.65	.61
OE	−.08	−.04	.10	**.31**	**.81**	**.80**	**.63**	**.43**	−.02	.01	−.02	−.07	.09	.00	.09	.17	.65	.61	.59	.55
PP	.28	**.46**	**.39**	**.46**	**.44**	.19	.17	.13	−.08	−.08	−.07	.13	.22	.20	.26	.23	.63	.50	.48	.58
OC	**.86**	**.60**	**.37**	**.64**	.03	−.06	−.01	.16	.13	−.04	.00	.15	−.24	.24	**.41**	−.14	.66	.50	.51	.58

Note. Factor loadings greater than 1 suggest Heywood cases. ^1^—Communalities represent the final estimates from the unrotated solution; WR—Word Reading; RC—Reading Comprehension; PD—Pseudoword Decoding; OF—Orthographic Fluency; DF—Decoding Fluency; ORF—Oral Reading Fluency; SP—Spelling; SC—Sentence Composition; EC—Essay Composition; AWF—Alphabet Writing Fluency; SWF—Sentence Writing Fluency; MPS—Math Problem Solving; NO—Numerical Operations; MFA—Math Fluency Addition; MFS—Math Fluency Subtraction; MFM—Math Fluency Multiplication; LC—Listening Comprehension; OE—Oral Expression; PP—Phonemic Proficiency; OC—Orthographic Choice; Columns represent age groups, 4 to 7, 8 to 11, 12 to 19, 20 to 50. **Bold** coefficients are greater than .30 **Bold** coefficients are greater than .30.

**Table A5:** Bifactor Solution with Four Specific Factors Across Age Groups.

	General				Factor 1				Factor 2				Factor 3				Factor 4				Communalities			
	4–7	8–11	12–19	20–50	4–7	8–11	12–19	20–50	4–7	8–11	12–19	20–50	4–7	8–11	12–19	20–50	4–7	8–11	12–19	20–50	4–7	8–11	12–19	20–50
WR	**.94**	**.88**	**−.87**	**.88**	.06	−.01	−.04	.03	.11	.14	.05	.05	.07	−.16	.14	.14	.16	−.14	.09	.16	.92	.83	.80	.82
RC	**.72**	**.69**	**−.66**	**.64**	−.23	**.35**	**.34**	**−.35**	.06	−.11	−.01	−.21	−.02	−.02	.01	−.01	−.06	.04	−.05	−.03	.58	.61	.55	.58
PD	**.84**	**.79**	**−.84**	**.85**	−.07	−.13	−.30	.03	.11	.10	−.02	.06	.10	−.11	.09	.07	−.02	−.23	.09	.04	.73	.71	.82	.73
OF	**.91**	**.83**	**−.83**	**.82**	.04	.04	.03	−.05	.02	−.01	**.39**	.11	.22	.03	−.01	.07	.08	**−.30**	.01	−.01	.89	.77	.83	.69
DF	-	**.81**	**−.79**	**.80**	-	−.17	−.23	.07	-	.01	**.33**	.28	-	.01	.00	−.01	-	**−.42**	.07	.25	-	.86	.80	.78
ORF	**.73**	**.73**	**−.66**	**.74**	.06	.01	−.07	−.22	−.06	−.05	**.38**	.05	.30	.09	−.05	.02	.12	−.30	−.10	.08	.65	.63	.59	.60
SP	**.83**	**.87**	**−.86**	**.90**	.18	−.21	−.07	.15	.00	.05	.04	−.01	−.05	−.07	.03	.00	−.14	.00	−.14	−.04	.74	.81	.77	.83
SC	**.69**	**.72**	**−.66**	**.51**	−.24	.06	.13	.05	.04	−.14	−.11	−.20	.04	.06	−.02	.01	−.19	**.36**	**−.30**	**−.37**	.57	.68	.56	.44
EC	-	**.61**	**−.54**	**.38**	-	−.29	.04	−.07	-	−.03	.02	−.19	-	.02	−.07	−.09	-	**.34**	**−.43**	**−.42**	-	.57	.49	.37
AWF	**.44**	**.39**	-	-	.10	.00	-	-	−.20	**−.68**	-	-	-.16	.10	-	-	-.20	.09	-	-	.31	.63	-	-
SWF	**.49**	**.37**	**−.37**	.20	−.02	.14	.07	−.25	−.19	−.29	.15	.13	.05	.24	−.19	−.13	**−.48**	−.06	.01	−.18	.51	.30	.20	.17
MPS	**.68**	**.70**	**−.76**	**.58**	**−.36**	**.31**	**.31**	−.04	−.15	.12	−.18	**−.58**	−.27	.30	−.24	**−.38**	.07	.13	.01	.00	.69	.71	.77	.81
NO	**.63**	**.65**	**−.67**	**.47**	−.04	.08	.24	.05	−.15	−.02	−.16	**−.61**	**−.46**	**.47**	**−.35**	**−.41**	.02	.18	−.16	−.01	.63	.68	.68	.77
MFA	**.59**	**.45**	**−.59**	**.52**	.02	−.01	−.05	−.06	**−.66**	−.03	.03	−.01	.00	**.69**	**−.67**	**−.76**	−.05	−.01	.02	.05	.79	.68	.80	.84
MFS	**.64**	**.54**	**−.64**	**.53**	−.08	.02	.01	−.04	**−.57**	.00	.02	.02	−.05	**.73**	**−.66**	**−.75**	.01	.02	.02	−.13	.75	.83	.84	.86
MFM	-	**.53**	**−.63**	**.35**	-	−.06	.01	.14	-	-.10	.02	-.07	-	**.66**	**−.63**	**−.76**	-	−.04	−.09	−.01	-	.73	.80	.73
LC	**.46**	**.56**	**−.60**	**.64**	**−.61**	**.53**	**.51**	**−.35**	−.01	.00	.01	−.20	−.02	−.02	.04	−.02	.00	−.04	.14	−.15	.58	.60	.64	.60
OE	**.60**	**.61**	**−.68**	**.68**	**−.54**	**.44**	**.36**	−.24	−.02	−.16	.01	−.19	−.02	.05	.00	−.01	−.14	.06	−.02	−.04	.66	.60	.59	.55
PP	**.74**	**.70**	**−.71**	**.73**	−.23	.01	−.02	−.24	.09	−.02	−.12	.01	−.10	−.09	.08	−.12	−.12	−.01	.05	.03	.63	.50	.53	.60
OC	**.77**	**.69**	**−.70**	**.74**	.03	−.14	−.02	.14	−.03	.09	.03	−.05	.04	−.07	.00	−.14	.23	−.04	−.15	.00	.65	.51	.51	.59
ω/ω.h	.92	.91	.92	.87	.03	.01	.01	.01	.02	.01	.00	.02	.00	.05	.04	.07	.00	.00	.00	.00				
H	.96	.96	.96	.96	.58	.53	.50	.38	.59	.52	.37	.59	.36	.77	.71	.82	.35	.46	.32	.36				
SS Loadings	8.37	9.00	9.26	8.20	1.02	.93	.84	.56	.93	.67	.52	1.02	.48	1.88	1.53	2.11	.47	.74	.41	.49				
Prop Tot Variance	.49	.45	.49	.43	.06	.05	.04	.03	.05	.03	.03	.05	.03	.09	.08	.11	.03	.04	.02	.03				
Prop Comm Var	.74	.68	.74	.66	.09	.07	.07	.04	.08	.05	.04	.08	.04	.14	.12	.17	.04	.06	.03	.04				

Note. WR—Word Reading; RC—Reading Comprehension; PD—Pseudoword Decoding; OF—Orthographic Fluency; DF—Decoding Fluency; ORF—Oral Reading Fluency; SP—Spelling; SC—Sentence Composition; EC—Essay Composition; AWF—Alphabet Writing Fluency; SWF—Sentence Writing Fluency; MPS—Math Problem Solving; NO—Numerical Operations; MFA—Math Fluency Addition; MFS—Math Fluency Subtraction; MFM—Math Fluency Multiplication; LC—Listening Comprehension; OE—Oral Expression; PP—Phonemic Proficiency; OC—Orthographic Choice; Columns represent age groups, 4 to 7, 8 to 11, 12 to 19, 20 to 50. **Bold** coefficients are greater than .30.

**Table A6:** Three Factor Oblique Solution Across Age Groups.

	Factor 1				Factor 2				Factor 3				Communalities ^1^			
	4–7	8–11	12–19	20–50	4–7	8–11	12–19	20–50	4–7	8–11	12–19	20–50	4–7	8–11	12–19	20–50
WR	**1.00**	**.88**	**.73**	**.89**	−.01	.16	.29	.05	−.05	−.16	−.13	−.09	.91	.82	.77	.79
RC	**.40**	.14	.08	.31	**.45**	**.73**	**.71**	**.54**	−.03	−.07	−.05	−.11	.58	.62	.55	.52
PD	**.76**	**.94**	**.85**	**.82**	.18	−.08	.00	.07	−.08	−.07	−.01	−.03	.73	.72	.71	.73
OF	**.97**	**.71**	**.75**	**.80**	−.05	.14	.11	.08	.01	.05	.06	−.04	.88	.72	.75	.69
DF	-	**.95**	**.99**	**1.00**	-	−.17	−.23	**−.34**	-	.07	.09	.16	-	.77	.79	.78
ORF	**.81**	**.61**	**.69**	**.66**	−.14	.09	−.06	.16	.06	.13	.12	−.03	.58	.57	.52	.57
SP	**.72**	**.88**	**.66**	**.74**	−.08	−.03	.24	.16	.21	.04	.03	.06	.68	.77	.75	.77
SC	**.34**	.20	.20	.14	**.44**	**.45**	**.50**	**.51**	.01	.16	.05	−.07	.54	.52	.48	.33
EC	-	**.46**	.24	−.02	-	−.01	.27	**.52**	-	.17	.13	.00	-	.32	.32	.26
AWF	.12	−.01	−	-	−.02	.31	-	-	**.44**	.20	-	-	.26	.20	-	-
SWF	.12	−.05	.17	.11	.13	.29	.06	.09	**.35**	.29	.25	.08	.29	.23	.18	.05
MPS	.00	.05	−.08	−.11	**.60**	**.51**	**.72**	**.71**	.26	**.32**	.28	**.31**	.63	.63	.76	.73
NO	.12	.04	−.14	−.21	.25	.24	**.56**	**.65**	**.38**	**.62**	**.44**	**.37**	.43	.66	.67	.63
MFA	−.02	−.03	.07	.13	−.11	−.09	−.12	−.02	**.94**	**.88**	**.92**	**.86**	.75	.67	.80	.81
MFS	.00	−.02	.04	.11	.06	−.03	−.01	.04	**.82**	**.93**	**.90**	**.82**	.72	.81	.84	.80
MFM	-	.07	.04	−.05	-	−.10	.01	−.07	-	**.87**	**.86**	**.91**	-	.73	.80	.74
LC	−.15	−.01	−.03	.27	**.86**	**.79**	**.81**	**.61**	−.07	−.13	−.11	−.12	.53	.51	.52	.55
OE	−.06	−.08	.10	**.36**	**.85**	**.84**	**.72**	**.50**	.00	.00	−.05	−.08	.65	.62	.58	.54
PP	**.36**	**.57**	**.46**	**.55**	**.49**	.22	**.33**	.21	−.02	−.06	−.06	.09	.61	.50	.48	.56
OC	**.73**	**.73**	**.47**	**.54**	−.02	−.03	.24	.13	.09	.00	.05	.21	.60	.50	.49	.56

Note. Factor loadings greater than 1 suggest Heywood cases. ^1^—Communalities represent the final estimates from the unrotated solution; WR—Word Reading; RC—Reading Comprehension; PD—Pseudoword Decoding; OF—Orthographic Fluency; DF—Decoding Fluency; ORF—Oral Reading Fluency; SP—Spelling; SC—Sentence Composition; EC—Essay Composition; AWF—Alphabet Writing Fluency; SWF—Sentence Writing Fluency; MPS—Math Problem Solving; NO—Numerical Operations; MFA—Math Fluency Addition; MFS—Math Fluency Subtraction; MFM—Math Fluency Multiplication; LC—Listening Comprehension; OE—Oral Expression; PP—Phonemic Proficiency; OC—Orthographic Choice; Columns represent age groups, 4 to 7, 8 to 11, 12 to 19, 20 to 50. **Bold** coefficients are greater than .30.

**Table A7:** Bifactor Solution with Three Specific Factors Across Age Groups.

	General				Factor 1				Factor 2				Factor 3				Communalities			
	4–7	8–11	12–19	20–50	4–7	8–11	12–19	20–50	4–7	8–11	12–19	20–50	4–7	8–11	12–19	20–50	4–7	8–11	12–19	20–50
WR	**−.92**	**.87**	**.85**	**.87**	.03	−.03	−.04	.18	−.06	.17	.13	−.13	.25	.17	.18	.06	.92	.82	.77	.81
RC	**−.72**	**.69**	**.66**	**.67**	−.26	**.36**	**−.33**	−.27	−.04	.01	.01	.00	.00	−.06	−.06	−.17	.58	.62	.55	.55
PD	**−.84**	**.79**	**.80**	**.85**	−.07	−.14	.16	.12	−.10	.12	.07	−.07	.06	.25	.19	.05	.73	.72	.71	.74
OF	**−.91**	**.83**	**.83**	**.82**	.06	.05	−.03	.02	−.03	−.03	−.02	−.06	.20	.29	**.32**	.11	.87	.77	.78	.69
DF	-	**.81**	**.78**	**.79**	-	−.15	.17	.26	-	−.01	−.01	.02	-	**.40**	**.42**	.29	-	.84	.82	.78
ORF	**−.73**	**.73**	**.67**	**.76**	.10	.03	.09	−.09	.02	−.09	−.05	−.03	.21	.27	.25	.08	.59	.62	.52	.58
SP	**−.84**	**.87**	**.87**	**.88**	.14	−.22	.09	.16	.04	.07	.05	.02	−.08	.01	.02	−.05	.73	.81	.77	.80
SC	**−.70**	**.73**	**.70**	**.49**	−.23	.07	−.03	−.11	−.05	−.07	.01	.02	−.14	**−.40**	−.26	−.25	.57	.70	.56	.32
EC	-	**.61**	**.59**	**.38**	-	**−.30**	.08	−.23	-	−.02	−.04	.10	-	**−.35**	−.20	−.24	-	.58	.40	.26
AWF	**−.44**	**.39**	-	-	.06	.11	-	-	.24	−.15	-	-	−.20	−.17	-	-	.29	.22	-	-
SWF	**−.53**	**.37**	**.37**	.22	.01	.18	−.07	−.30	.14	−.26	−.19	.12	**−.39**	−.01	.12	.13	.45	.24	.19	.17
MPS	**−.64**	**.69**	**.75**	**.58**	**−.44**	.26	**−.32**	−.04	.24	−.28	−.23	**.38**	.06	−.08	−.15	**−.55**	.66	.62	.75	.78
NO	**−.58**	**.65**	**.69**	**.46**	−.16	.07	−.18	.04	.26	**−.47**	**−.33**	**.42**	−.02	−.16	−.25	**−.60**	.43	.67	.68	.76
MFA	**−.58**	**.45**	**.59**	**.52**	.03	−.02	.04	−.02	**.63**	**−.69**	**−.67**	**.75**	−.05	.02	.05	.01	.74	.68	.80	.83
MFS	**−.63**	**.54**	**.64**	**.53**	−.09	.01	−.02	−.09	**.60**	**−.72**	**−.66**	**.75**	.02	.00	.03	.01	.76	.82	.84	.85
MFM	-	**.53**	**.65**	**.34**	-	-.05	.02	.11	-	**−.67**	**−.62**	**.78**	−	.02	−.04	−.08	-	.73	.80	.73
LC	**−.44**	**.56**	**.58**	**.67**	**−.60**	**.51**	**−.56**	**−.35**	.02	.02	.02	.01	.02	.04	.03	−.18	.56	.57	.64	.61
OE	**−.60**	**.62**	**.68**	**.70**	**−.53**	**.46**	**−.35**	−.19	.02	−.07	.01	.01	−.10	−.10	−.04	−.17	.65	.61	.59	.55
PP	**−.74**	**.70**	**.69**	**.74**	−.27	.01	−.04	−.13	-.05	.08	.07	.11	−.09	.01	.03	.04	.63	.50	.48	.58
OC	**−.75**	**.68**	**.71**	**.73**	.00	−.16	.05	.16	.07	.07	.01	.16	**.30**	.06	−.02	−.08	.65	.50	.51	.58
ω/ω.h	.90	.91	.92	.87	.03	.01	.01	.00	.03	.05	.03	.07	.00	.00	.00	.02				
H	.96	.96	.96	.96	.60	.53	.49	.39	.59	.77	.71	.82	.34	.46	.41	.58				
SS Loadings	8.23	9.00	9.29	8.23	1.10	.01	.76	.59	.99	.05	1.49	2.14	.48	.00	.63	1.02				
Prop Tot Variance	.48	.45	.49	.43	.06	.53	.04	.03	.06	.77	.08	.11	.03	.46	.03	.05				
Prop Comm Var	.76	.71	.76	.69	.10	.84	.06	.05	.09	.93	.12	.18	.04	.84	.05	.08				

Note. WR—Word Reading; RC—Reading Comprehension; PD—Pseudoword Decoding; OF—Orthographic Fluency; DF—Decoding Fluency; ORF—Oral Reading Fluency; SP—Spelling; SC—Sentence Composition; EC—Essay Composition; AWF—Alphabet Writing Fluency; SWF—Sentence Writing Fluency; MPS—Math Problem Solving; NO—Numerical Operations; MFA—Math Fluency Addition; MFS—Math Fluency Subtraction; MFM—Math Fluency Multiplication; LC—Listening Comprehension; OE—Oral Expression; PP—Phonemic Proficiency; OC—Orthographic Choice; Columns represent age groups, 4 to 7, 8 to 11, 12 to 19, 20 to 50. **Bold** coefficients are greater than .30.
